# Porphyrin Photosensitizers Grafted in Cellulose Supports: A Review

**DOI:** 10.3390/ijms24043475

**Published:** 2023-02-09

**Authors:** Carlos J. P. Monteiro, Maria G. P. M. S. Neves, Cristina Nativi, Adelaide Almeida, Maria Amparo F. Faustino

**Affiliations:** 1 LAQV-Requimte and Department of Chemistry, University of Aveiro, 3010-193 Aveiro, Portugal; 2 Department of Chemistry “Ugo Schiff”, University of Florence, via della Lastruccia, 3-13, 50019 Sesto Fiorentino, Italy; 3 CESAM and Department of Biology, University of Aveiro, 3810-193 Aveiro, Portugal

**Keywords:** antimicrobials, photodynamic therapy, cellulose functionalization, cellulose-based material, porphyrin, photosensitizer, bacteria, support

## Abstract

Cellulose is the most abundant natural biopolymer and owing to its compatibility with biological tissues, it is considered a versatile starting material for developing new and sustainable materials from renewable resources. With the advent of drug-resistance among pathogenic microorganisms, recent strategies have focused on the development of novel treatment options and alternative antimicrobial therapies, such as antimicrobial photodynamic therapy (aPDT). This approach encompasses the combination of photoactive dyes and harmless visible light, in the presence of dioxygen, to produce reactive oxygen species that can selectively kill microorganisms. Photosensitizers for aPDT can be adsorbed, entrapped, or linked to cellulose-like supports, providing an increase in the surface area, with improved mechanical strength, barrier, and antimicrobial properties, paving the way to new applications, such as wound disinfection, sterilization of medical materials and surfaces in different contexts (industrial, household and hospital), or prevention of microbial contamination in packaged food. This review will report the development of porphyrinic photosensitizers supported on cellulose/cellulose derivative materials to achieve effective photoinactivation. A brief overview of the efficiency of cellulose based photoactive dyes for cancer, using photodynamic therapy (PDT), will be also discussed. Particular attention will be devoted to the synthetic routes behind the preparation of the photosensitizer-cellulose functional materials.

## 1. Introduction

Functionalized cellulose-based materials have attracted special attention from the scientific community, mainly in the field of biological and medical sciences [1,2,3]. As cellulose demonstrates a carbohydrate nature, it has inherent compatibility with biological tissues and, consequently, possesses unique applicability with respect to its bioavailability, biocompatibility, and biodegradability considerations [3,4].

Cellulose is the most abundant natural biopolymer, considered a valuable starting material for developing new materials from renewable resources [5,6,7], with an estimated annual production of 7.5 × 10^10^ tons [8]. Cellulose is available from several natural sources, mainly from trees and green plant cell walls, but also from some types of algae and tunicates or bacteria [9].

**Figure 1:**
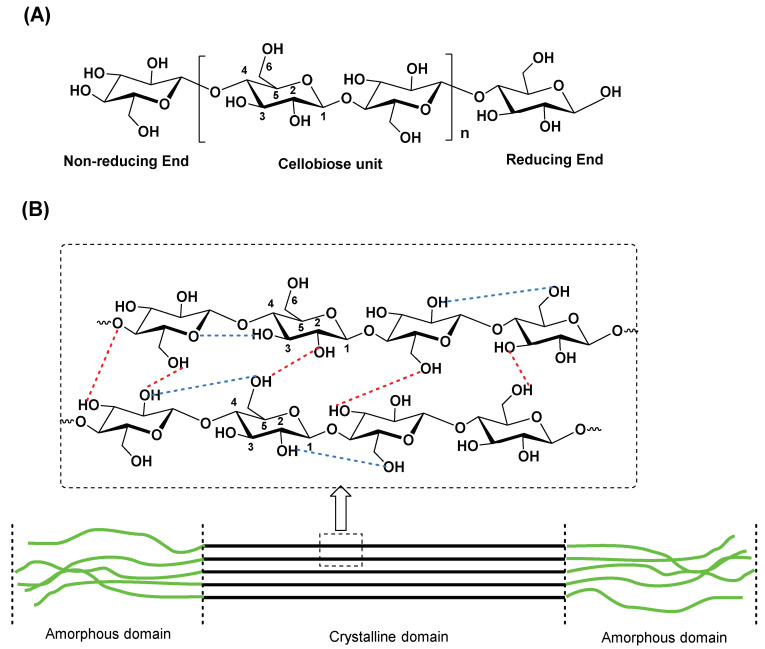
Structure of cellulose: (**A**) Molecular structure of cellulose with cellobiose as a repeating unit and (**B**) inter (red dotted line) and intra molecular hydrogen bonds (blue dotted line) interactions between cellulose chains within the crystalline region of the cellulose microfibrils.

Cellulose in higher vascular plants consists of the xylem tissues, which are known as wood, where cellulosic microfibrils are embedded in a matrix of hemicellulose (an amorphous and complex mixture of linear and branched polysaccharides) and lignin, an unstructured phenolic polymer, randomly branched and crosslinked with cellulose. In nature, this β-*O*-(1 → 4) homopolymer does not occur as a single chain but as multiple chains (Figure 1A,B) [5,6]. Lignin is commonly derived from wood and one of most abundant organic compounds on Earth, after cellulose and chitin [10,11].

The ribbon-like character observed for cellulose biopolymer chains, are tightly bundled by van der Waals and hydrogen bonds, allowing adjacent cellulose chains to fit closely together (Figure 1B), assembling three-dimensional networks known as microfibrillated cellulose (MFC) [12]. In these cellulose fibrils, some regions can be found with highly ordered chains (crystalline domain) and others with chains randomly and highly disordered (amorphous domain) (Figure 1B) [13]. The separation or extraction of the crystalline regions from amorphous segments is usually performed by treating the cellulose fibres with an acid (e.g., sulfuric, nitric, or hydrochloric acids), leading principally to the disruption of amorphous regions while leaving the crystalline segments intact. From this disintegration, either cellulose nanofibers (CNF) or nanocrystalline cellulose (NCC) can be obtained, the latter also known as cellulose nanocrystals (CNCs). These CNCs possess unique properties, such as high surface area, high mechanical strength, and a liquid crystalline nature [14,15,16,17] with applications in several fields, such as biomedicine [18,19], wastewater treatment [20,21], biobased adsorbents of metals [22,23,24], energy [25,26], and photonic platforms [27,28]. 

**Figure 2:**
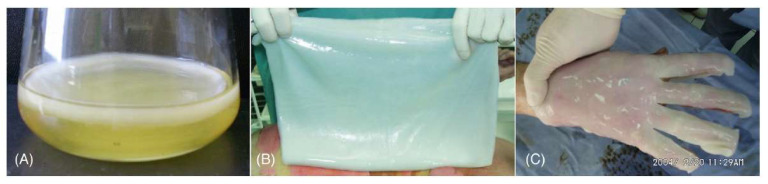
Medical and cosmetic applications of bacterial nanocellulose: (**A**) cellulose layer grown on the top of bacterial culture; (**B**) wound dressing prepared form purified cellulosic membrane; (**C**) application of cellulose hydrogel patch (clinical context) in a burn injury. Reprinted with permission from Ref. [3], 2016, Elsevier Ltd.

As mentioned before, bacteria, are also able to synthesize cellulose in aqueous culture media. Bacterial cellulose (BC), firstly identified in 1886 by A.J. Brown [29] appears as a jelly-like translucent polymer of excellent chemical purity. Under special culturing conditions, BC can be produced extracellularly, in the form of nanofibers, through fermentation mediated by Gram-(−) bacterial cultures of *Gluconacetobacter*, *Acetobacter*, *Agrobacterium*, *Achromobacter*, *Aerobacter*, *Sarcina*, *Azobacter*, *Rhizobium*, *Pseudomonas*, *Salmonella* and *Alcaligenes*. The most efficient bacterial strain for BC production belongs to the *Gluconacetobacter* type [30,31]. During biosynthesis, BC forms a thick gelatinous membrane called pellicle, controllable through the alteration of fermentation parameters, constituted by a random microfibrillar network of cellulose chains aligned in parallel, scattered among amorphous regions that occupy 90% of the materials total volume (Figure 2A) [32].

Bacterial cellulose has the same molecular formula as plant cellulose, but it is characterized by peculiar three-dimensional porous net-work structures with a high degree of polymerization (up to 8000), high crystallinity (70–80%), high water content (up to 99%), and high mechanical stability, [33]. Depending on the BC biofabrication approach (i.e., fermentation process, type of strain, carbon source, and additives), BC shape and supramolecular structure can give rise to different film forms, sizes or thicknesses, being precisely tailored for diverse applications [31]. The inclusion of additives in the nutrient media components during biosynthesis can influence the assembly and microstructure of BC, including the crystallinity, crystalline polymorphism, crystal size, and ribbon width [34]. The BC feature properties, such as controlled porosity, biocompatibility biodegradability, flexibility, ease of production, and absence of contaminants (e.g., lignin and hemicellulose), have raised the interest of academic and pharmaceutical industry communities for its utilization as a competitive product. The high biocompatibility of this hydrogel material has a high impact on its ubiquitous applications in the medical field [3,35]. In fact, this natural polymer is being successfully utilized in the reconstruction and regeneration of tissues as artificial skin (Figure 2B,C) [35], in vivo implants [36], artificial blood vessels [37], wound healing scaffolds [38], hemostatic materials [39], energy storage [40], and in electronic devices [41]. In addition to CNC and BC, cellulose fibres, as fabrics or as paper sheets present unique mechanical and physical properties to be applied as functional materials for medical applications. According to the literature, cellulose fibres are among the most interesting basic materials for functionalization with antimicrobial agents [4]. The grafting of antimicrobials, as photosensitisers or biocides, would confer exclusive features for medical applications, such as wound healing patches to treat infected diabetic foot/leg ulcers or regeneration of damaged tissue in burn wounds [7,42,43]. The functionalization/immobilization of cellulose fibres with photoactive compounds can be an advantage in the mitigation of nosocomial pathogenic microorganisms. Furthermore, functionalized cellulose fibres with photoactive antimicrobials have also been considered for protective clothing (e.g., surgical masks, caps, gowns) and hospital linen, providing the mitigation of harmful microorganism transmission and spreading of secondary infections. All this can minimize risks for developing a serious illness, to prolong the healing of patients, and consequently reduce economic losses and extra costs to the health.

Antimicrobial drugs have had a tremendous impact on modern medicine and have been viewed as a *panacea* for several infectious diseases, over the last 80 years [44,45].

Sulfonamides or sulfa drugs were the first effective synthetic drugs used systematically against a broad bacterial spectrum [46,47]. Since their discovery in 1935 [48,49], that microbial resistance to antibiotics is a major concern. The widespread, excessive dispensing and irresponsible use of antibiotics, their dissemination in the environment, associated with the globalization of pathogenic microorganism transmission have resulted in the development of resistant bacterial strains [50,51,52].

It was estimated that, if no urgent actions are taken shortly, antimicrobial resistance (AMR) will have a full impact in terms of human and economic losses: between 2015 and 2050, 100 × 10^12^ USD would be lost, and by 2050 microbial resistance will kill 10 × 10^6^ people per year, outweighing the death caused by cancer [53]. In fact, a recent study confirms these predictions, with 4.95 million (3.62–6.57 million) deaths associated with bacterial AMR in 2019 was estimated, showing that AMR bacteria represent a health problem with a magnitude at least as large as major diseases, such as HIV and malaria, being a problem in all world regions [54].

Recent strategies have focused on the development of novel antimicrobial treatment options and alternative therapies. So, the new alternative strategies must encompass different mechanism of action, circumventing resistance issues of conventional antimicrobials.

In the last few years, the development and biological assessment of new photosensitizers (PS) for antimicrobial photodynamic therapy (aPDT) were accompanied by their immobilization in different supports considering applications, such as wound disinfection and healing, sterilization of medical devices and surfaces in different contexts (industrial, household and hospital), or the prevention of microbial contamination in packaged food [42,43,55,56,57,58,59,60,61,62,63]. Photosensitizers for aPDT can become more efficacious when adsorbed, entrapped, or linked to cellulose-like supports [64,65,66,67,68,69]. In addition, these cellulose-based systems provide an increase in the surface area, and to improve the targeted drug delivery [70,71], can be used to develop packaging materials with better mechanical strength, barrier, and antimicrobial properties [72,73].

aPDT is a non-antibiotic therapeutic approach that like PDT involves the combination of photoactive dyes or PS, harmless visible light, and dioxygen (O_2_) [44,64,65,74,75]. The photoactive drug (PS), is a non-toxic molecule in the dark, but when electronically excited can transfers an electron to O_2_ or other electron acceptors leading to the formation of reactive oxygen species (ROS) such as superoxide anions (O_2_^−●^) and other radicals (e.g., HO^●^, type I reaction), or transfers its electronic energy to ground-state dioxygen (^3^O_2_) generating singlet oxygen (^1^O_2_) (type II reaction) [76,77,78,79]. These ROS can oxidize biomolecules, such as DNA, proteins, and lipids, causing oxidative cellular damage, leading to pathogen inactivation [80].

Since the photodynamic action is a multi-target mechanism, it is highly unlikely that this approach causes microorganisms to develop resistance, as happens with conventional antimicrobials that generally work on a one-target principle [81,82,83,84,85]. Furthermore, photodynamic action can impair the activity and/or production of many virulence factors, leading to the decrease of bacterial pathogenicity, contrasting antibiotic therapy, which can induce the build-up of virulence factors, leading to their release upon antibiotic treatment [86,87,88,89].

Despite the known aPDT advantages and its efficacy, there is still room for new improvements, maximizing the full potential for translational research and development.

This review will report on recent advances in the development of a diversity of porphyrin photosensitizers (PS) supported on cellulose/cellulose derivative materials to achieve effective antimicrobial materials. In addition to aPDT, cellulose-based hybridized carriers have been found in the literature for other biomedical applications. Thus, in this review paper, we have considered a few examples of cellulose multifunctional nanostructures for application in cancer photodynamic therapy (PDT). The improvement of treatment outcomes using cellulose as PDT photosensitizer carriers will also be described and discussed.

Particular attention will be devoted to the synthetic routes behind the preparation of the photosensitizer-cellulose functional materials. 

So, after a brief discussion concerning the chemical modifications of cellulose to allow the mobilization of the photoactive dye, this review is organized considering first the porphyrin and analogues of natural origin and then the synthetic counterparts according to their charges.

## 2. Functionalization of Cellulose Supports

Their unique properties, availability, and surface chemical flexibility make cellulose an attractive and versatile raw material for well-known features and uses in many industrial applications [59,72,90]. Cellulose is being used in dietary fibres and food additives [91], personal care products [3], templates for electronic components [41,92], separation membranes [93,94], batteries [40], and supercapacitors [95]. Nowadays, cellulose is also being recognised as an important component for more advanced purposes, such as photocatalytic materials [96,97,98,99], biomedical implants [100,101], pharmaceuticals [4,102], drug delivery [3,103], and for the development of antibacterial biomaterials [38,60,104,105,106,107].

**Figure 3:**
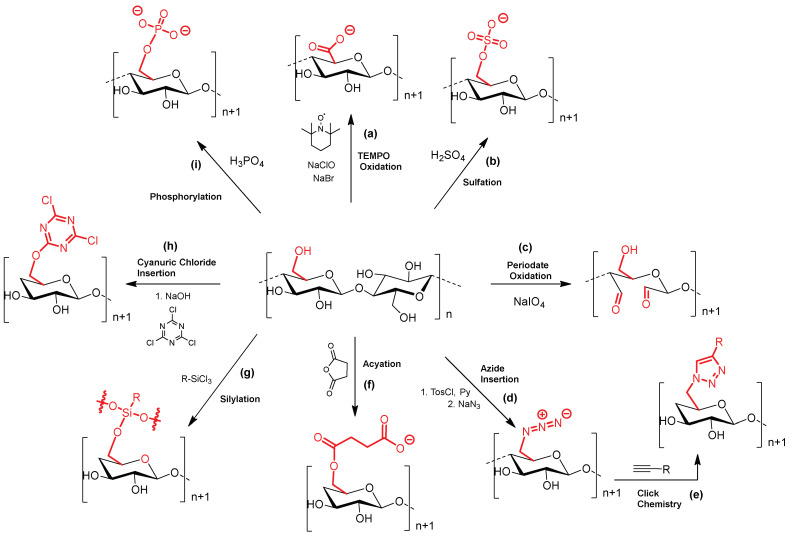
Common chemical modifications of cellulose discussed in the current review.

Most of these applications are strongly dependent on specific transformations of the cellulose backbone. Many hydroxyl groups present in the cellulose structure can be chemically modified, providing a platform with functional features suitable for a plethora of applications. The most common modifications are: (i) grafting of new active units for further functionalization via covalent linkages; or (ii) modification of the surface charge density for electrostatic/noncovalent interactions with surfactants, polymers, and counter ion salts. Figure 3 displays the cellulose chemical modifications that will be discussed in the following sections of the current review.

One of the most common approaches used to obtain highly negative charged cellulose sulphate is through cellulose degradation mediated by sulphuric acid, affording CNCs decorated with sulphate esters (Figure 3, path b) [108]. Although the presence of sulphate esters (obtained by esterification of some hydroxyl groups) compromises the CNCs thermal stability, they are useful for the formation of stable colloidal suspensions by electrostatic interaction between charged surfactants, such as cetyltrimethylammonium bromide (CTAB) [5,6].

The phosphorylation of cellulose conducts to the formation of phosphate esters and consequently to negatively charged cellulose (Figure 3, path i), accountable for important applications [109,110]. For instance, the extraordinary binding ability of phosphate groups to calcium ions or growth factors is being explored in different biological applications [111]. In addition, these functional materials can be used to make materials with flame-resistant and flame-proof properties [112], to be used as supercapacitors [95] or in environmental applications [93,94].

TEMPO (2,2,6,6-tetramethylpiperidine-1-oxyl radical) can also be used to introduce negative charges on cellulose surface (Figure 3, path a), by converting primary hydroxymethyl groups into carboxylic residues [113,114,115]. The oxidation of cellulose with a catalytic amount of TEMPO, NaBr, and NaClO selectively transforms cellulose C_6_ primary hydroxyl groups into aldehyde groups and carboxyl groups [111,116].

Periodate-mediated oxidation of cellulose is a different approach to make carbonyl-modified cellulose derivatives. Unlike TEMPO, the reaction of cellulose with periodate leads to the cleavage of C_2_ and C_3_ bond of glucose monomer units affording 2,3-dialdehyde cellulose (DAC) (Figure 3, path c) [117]. These aldehyde functionalities in DAC can be further converted into sulfonic acids, carboxylic acids, and imines [107,118]. Moreover, the treatment of cellulose with periodate makes cellulose more flexible, with an improvement in dispersibility.

Organosilyl units have also been used to decorate cellulose surface [119,120]. In general, these entities are introduced by making use of silicon reactants bearing hydrolysable groups (typically alkoxy, acyloxy, amine, or chlorine) that can react with hydroxyl groups on cellulose (Figure 3, path g). The non-hydrolysable unit typically holds a moiety that can react with a biologically active molecule [121,122].

The reaction with 2,4,6-trichloro-1,3,5-triazine (also known as cyanuric chloride) is a good approach to deliver a very flexible platform to covalently couple active molecules to cellulosic substrates (Figure 3, path h) [73,123,124,125]. The stepwise substitution of chlorine atoms with nucleophiles (such as amines, phenols, and thiols) is facile, proceeds under smooth reaction conditions, and can be controlled by temperature since the reactivity decreases with the increase in the number of substituents linked to the cellulose framework [126,127].

Cu(I)-catalyzed Huisgen 1,3-dipolar cycloaddition (*Click-Chemistry* reaction) was also considered an attractive approach to covalently conjugate functional molecules onto the cellulose surface. In this *click* reaction, the coupling of an azide moiety, previously introduced (Figure 3, path d) with a terminal alkyne, leads to the formation of a stable 1,2,3-triazole ring (Figure 3, path e) [128,129]. In this approach, cellulose primary hydroxyl groups are first converted into tosylate (-OTs) leaving groups, followed by their nucleophilic substitution with sodium azide (NaN_3_) in dimethylformamide (DMF), affording cellulose displaying azide functionalities (Figure 3, path d). A *click* reaction involving a molecule bearing a propargylic unit can then produce cellulose bearing triazole moieties (Figure 3, path e). However, this strategy can proceed in a different way i.e., the cellulose support can be first functionalized with the alkynyl unit via propargyl bromide and then the molecule to be appended can contain the azide functionality [130].

The acylation of hydroxyl groups present at cellulose surface is another widely used modification [131]. This functionalization can occur through the reaction with acids or with succinic, maleic, or phthalic anhydrides (Figure 3, path f) [132]. With these cyclic anhydrides, the functionalization results in a pendant carboxylic moiety attached to the cellulose via a covalent ester bond, providing reactive moieties for further functionalization [133].

## 3. Synthetic Access to Cellulose-Based Photosensitizers

### 3.1. Natural Porphyrin and Reduced Derivatives: Protoporphyrin IX and Hydroporphyrins

#### 3.1.1. Protoporphyrin IX Derivatives

Tetrapyrrolic macrocycles, e.g., protoporphyrin IX (PPIX), play a key role in life [134,135]. PPIX can chelate transition metals, affording metalloporphyrins, which accomplish a variety of biological functions. For instance, the complexation of PPIX with Fe(II) forms Heme, a constituent of hemoproteins with significant roles in dioxygen transportation, cellular oxidations and reductions, electron transportation, and drug metabolism. PPIX has some biological functions of its own, and PPIX-based compounds have been used as PS for cancer and aPDT [136].

Considering the photobiological features of PPIX, the development of cellulose based photoactive hybrid materials has attracted the attention of several research groups.

**Figure 4:**
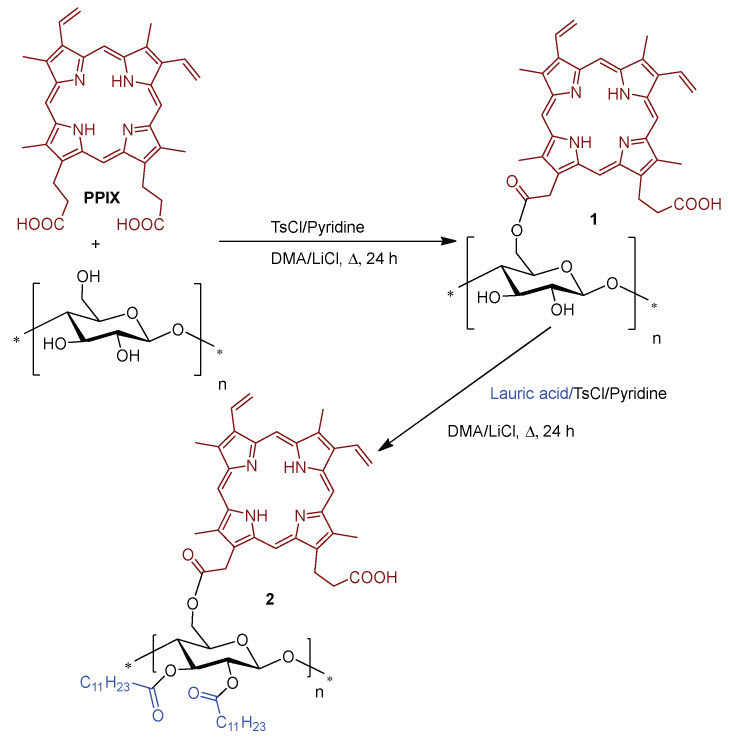
Synthetic approach for preparation of photobactericidal films **2** by *“one-pot, two-step”* esterification of cellulose with PPIX and lauric acid (adapted from Ref. [137]).

In 2006, Krausz and co-workers [137] developed photobactericidal films **2** obtained by “one-pot, two-steps” esterification of cellulose with PPIX and lauric acid (Figure 4). The first step comprises the esterification of cellulose with PPIX using tosyl chloride (TsCl) plus pyridine to activate the primary hydroxyl groups, followed by a second step, under similar conditions, concerning the esterification of the remaining OH groups present on the cellulose-appended PPIX with the lauric acid.

The biological results on the inactivation of *Escherichia coli* and *Staphylococcus aureus* strains revealed that the observed antibacterial activity, upon 24 h of light irradiation (irradiance of 1.7 mW cm^−2^) of the prepared plastic films **2**, was reliant on the porphyrin grafting percentage, attaining sterile areas (no bacteria growth) under the plastic film disks **2**. In the absence of PPIX, the films did not hamper a massive growth of bacteria. The authors stated that these photoactive materials could be applied in industrial, household, and medical environments, and more generally in areas that would benefit from permanent and efficient surface disinfection.

In 2018, Wei [138] research group prepared a photoactive material comprising PPIX covalently grafted onto a bacterial cellulose (**BC**) surface via diamine spacer arms with different chain lengths. Thus, three diamine spacers, with backbone chain lengths of four (ethylenediamine), ten [1,2-bis(2-aminoethoxy)ethane], and 14 [1,4-bis(3-aminopropoxy)butane] atoms were applied in order to analyze their influence on BC surface functionalization and subsequent bacterial photoinactivation performance.

**Figure 5:**
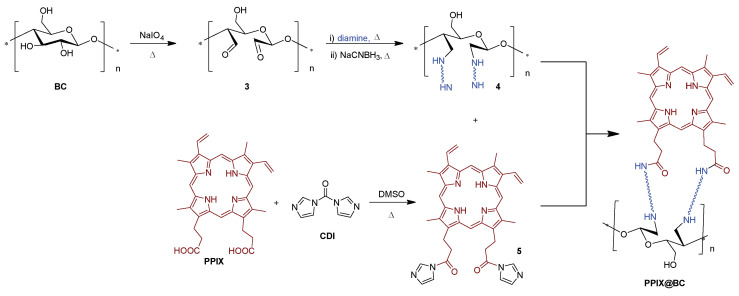
Preparation of PPIX covalently grafted onto a bacterial cellulose surface (**3**) via diamine spacer arms with different chain lengths, (adapted from Ref. [138]).

Oxidation of **BC** was mediated by sodium periodate (NaIO_4_) in an aqueous solution and then treated with 1,2-ethanediol overnight to remove the excess of periodate, giving material **3** (Figure 5). The incorporation of amines on **3** was carried out by the “one pot, two steps” method, consisting of the cellulose membrane immersion in a solution of the respective diamine (ethylenediamine, 1,2-bis(2-aminoethoxy)ethane and 1,4-bis(3-aminopropoxy)butane) for 12 h, followed by the addition of the reducing agent sodium cyanoborohydride. The reaction continued for additional 24 h. Finally, after washing steps, the material functionalized with NH_2_ groups **4** was coupled with the PPIX derivative **5** previously activated with carbonyldiimidazole (CDI), (Figure 5) giving the photoactive material **PPIX@BC**. After 24 h, the remaining free PPIX was thoroughly washed, and the resulting membrane dried under vacuum.

Antibacterial efficacy was evaluated under white light irradiation (Xenon lamp, 30 min, λ > 420 nm) against both *S. aureus* and *E. coli* strains as a function of the diamine spacer unit. The best inactivation results were obtained for the **PPIX@BC** membranes with diamine spacers with backbone chain lengths of 10 atoms [1,2-bis(2-aminoethoxy)ethane], attaining 99.999% (5 log of reduction in CFU mL^−1^) of photoinactivation efficiency against *E. coli* meanwhile *S. aureus* was inactivated by 98.5% (~1.5 log reduction in CFU mL^−1^) by the same photosensitizing material. The results obtained are very interesting since the photodynamic effect of PPIX immobilized on **BC** is higher for the Gram-(−) bacterium *E. coli* than that of the non-immobilized PPIX under similar experimental conditions. The authors explained these results due to the incomplete grafting of PPIX onto the aminated BC surface, leading to the pronation (in buffer) of free primary amine groups on BC-spacer -NH_2_, giving -NH_3_^+^, that would result in a positively charged surface. As a consequence, negatively charged bacteria would adhere onto the supported surface. Therefore, it would lead to a stronger interaction of *E. coli* to the surface, bringing it in closer proximity to the PS molecules and to the site of ^1^O_2_ formation, leading to higher photodynamic inactivation.

The reusability of **PPIX@BC** membranes was explored through the repetition of aPDT assays. A gradual decreasing of the antibacterial efficacy was observed for *E. coli*, with aPDT efficiency reduced from the original 99.999% (5 log of reduction, 1st cycle) to approximately 45% for the 4th cycle. In spite of the need to improve the reusability of materials, the aforementioned work provides a powerful strategy for efficient Gram-(−) bacteria photodynamic inactivation.

**Figure 6:**
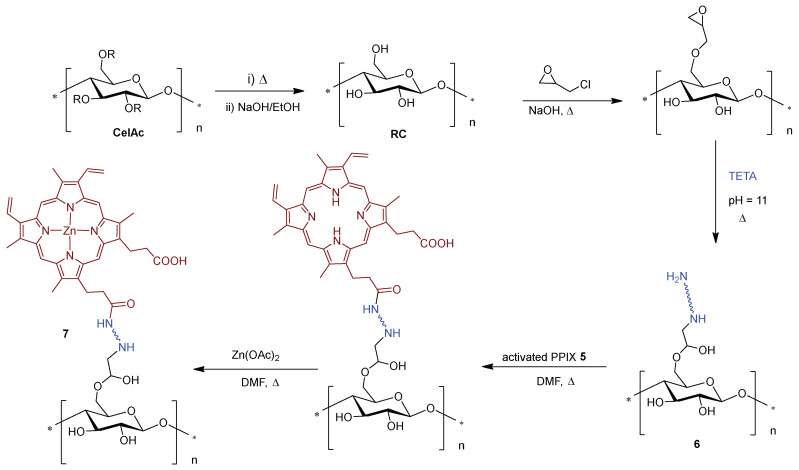
Covalent grafting of activated PPIX **5** using epichlorohydrin functionalized cellulose nanofibers **6** (adapted from Ref. [139]).

The promising results of aPDT accomplished by Wei and co-authors motivated the same authors to extend the strategy to the preparation of an improved photoactive material [139] with enhanced mechanical properties, high efficacy under low light conditions, and robustness towards photobleaching and reusability. The synthetic route employed was slightly different from the previous one: cellulose acetate (**CelAc**) was electrospun to produce nanofibers, thermally treated to enhance mechanical strength, and finally hydrolyzed to produce regenerated cellulose (**RC**) nanofibers that possessed a high surface area and nanofibrous structure. Covalent grafting of the activated PPIX **5** on epichlorohydrin/triethylenetetramine (TETA) functionalized cellulose **6**, followed by zinc(II) complexation of porphyrinic macrocycle, afforded material **7** with a loading of 412 nmol Zn(II)PPIX/mg material (Figure 6). Antibacterial efficacy was evaluated against *S. aureus* (ATCC 6538) and *E. coli* (ATCC 8099), with results achieving detection limit inactivation (99.999%, >5 log of reduction) for both bacteria after 20 min of white light illumination (Xe lamp, 500 W, λ > 420 nm, 10 cm distance between lamp and sample). No statistically significant loss in antibacterial activity was observed when using nanofibers that had been photo-aged with 5 h of pre-illumination to simulate the effects of photobleaching. Taken together, the results suggest that such systems may employ cost-effective neutral PS paired with a wide range of polymeric scaffolds for the development of robust and durable self-sterilizing materials based on a photodynamic mode of action.

**Figure 7:**
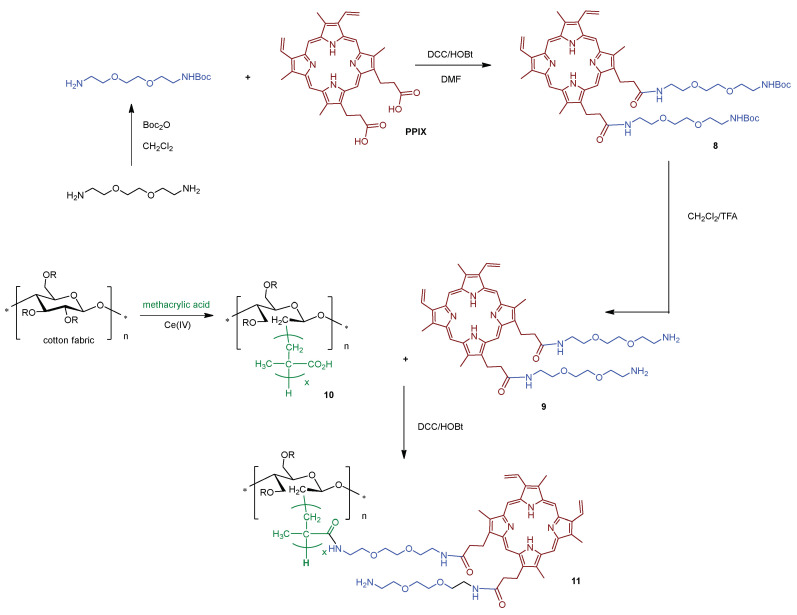
Synthetic strategy adopted by Sol: aminoethyleneglycol spacer arms modified PPIX **9** in cotton/polymethacrylic supports **10** (adapted form Ref. [140]).

In 2018, Sol research group [140] developed a new antimicrobial material consisting of PPIX amino derivative, **9** bearing aminoethyleneglycol spacer arms immobilized in cotton/polymethacrylic support **10** (Figure 7). The authors polymerized methacrylic acid on cotton material, mediated by Ce(IV) as a redox radical polymerization system, affording the support **10**. Aminoethyleneglycol spacer arms were appended on PPIX to facilitate its grafting to the carboxylic acid groups present at the polymethacrylic acid polymerized /cotton fabric. The first step involved the functionalization of PPIX with 1,2-bis(2-aminoethoxy)ethane monoprotected with *tert*-butyloxycarbonyl (Boc) in the presence of dicyclohexylcarbodiimide (DCC)/1-hydroxybenzotriazole (HOBt) giving **8**. After acid deprotection of the amino groups, the desired PPIX amino derivative **9**, obtained in quantitative yield, was successfully attached to **10** using classical peptide linkage conditions, yielding the material **11** (Figure 7). The photodynamic activity of photoactive cotton was evaluated against *S. aureus* and *E. coli*. Experimental data show that this cotton textile **11** bearing the PPIX derivative exerts only a photo-antibacterial effect against *S. aureus*, with inactivation of 99.9% (3 log reduction in CFU mL^−1^) after 24 h of white light irradiation (total light dose 9.5 J cm^−2^). Concerning Gram-(−) bacterium *E. coli*, the photosensitizing material did not show any photoactivity.

Zhang research group [69] prepared a water-soluble hybrid photoactive material introducing PPIX and quaternary ammonium salt (**QAS**) groups onto a cellulose backbone by means of an esterification reaction.

**Figure 8:**
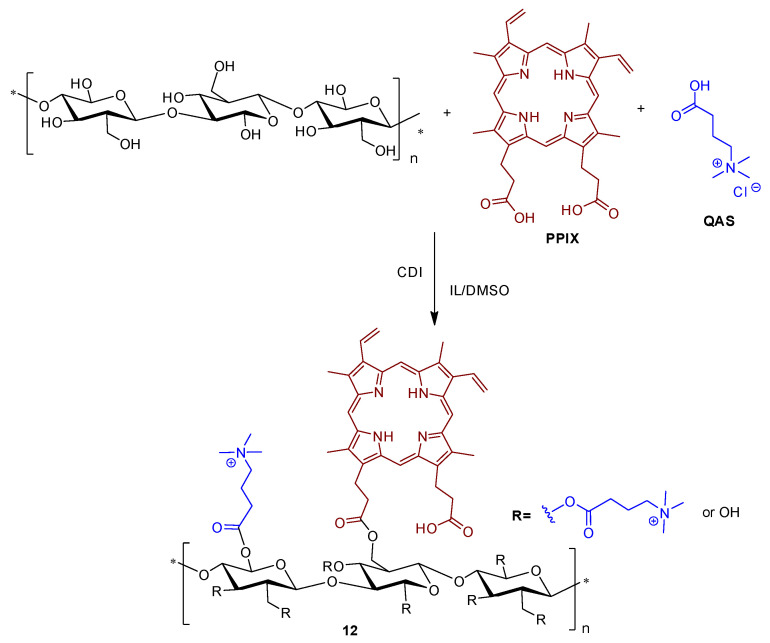
Strategy for preparation of water-soluble hybrid photoactive material **12** containing PPIX and **QAS** groups onto a cellulose support (adapted from Ref. [69]).

The introduction of positively charged quaternary amines led to a synergistic effect, increasing water-solubility and electrostatic repulsion between quaternary positive charge groups, which effectively inhibits the π–π stacking of immobilized PPIX, thus enhancing ROS yield (Figure 8).

The inhibition efficiencies of material **12** toward the drug-resistant *E. coli* and *S. aureus* were respectively 84% (~0.79 log of reduction) and 98% (~1.70 log of reduction) upon 1 min of white light irradiation at an irradiance of 40 mW cm^−2^. After 2 min of white light irradiation, the inhibition efficiencies toward *E. coli* and *S. aureus* increased to 93% (~1.15 log of reduction) and >99% (>2 log of reduction), respectively.

**Figure 9:**
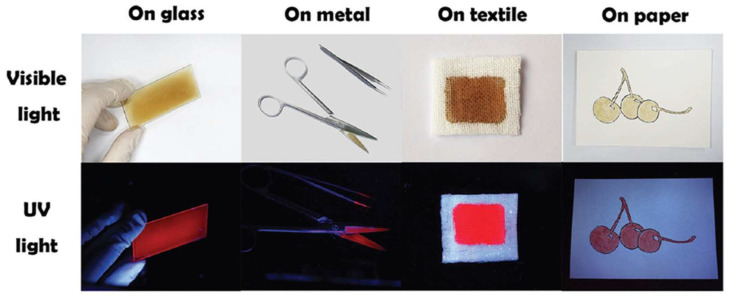
Images of cellulose-based PS **12** on different substrates. Reprinted with permission from Ref. [69]. 2008, Elsevier.

The applicability and broad use of the photoactive cellulose-PPIX material **12** were proved in different substrates (e.g., slide glass, scissors, tweezers, gauze, and paper) and in personal bioprotective equipment, e.g., face masks, protective suits, and gloves (Figure 9).

In these assays, two areas on a face mask, a protective suit, and a glove, were chosen and one of these areas was coated with the photoactive cellulose-PPIX material **12**. The selected areas were inoculated with a bacterial suspension and irradiated with white light and sunlight. Then, bacteria survival rates were assessed in these areas. There were almost no colony forming units of *E. coli* and *S. aureus* on the coated areas of the face mask, protective suit, and glove after 15 min of white light and sunlight irradiation, corresponding to around 3–4 log of bacterial inactivation. Indeed, the water-soluble and nontoxic cellulose-based PPIX material **12** exhibited excellent antibacterial activity to drug-resistant bacterial strains with promising applications as wearable and robust sunlight-driven antibacterial coatings.

**Figure 10:**
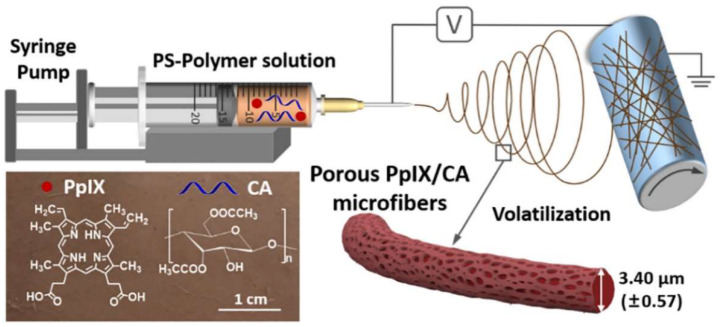
Scheme for the preparation of the porous **PPIX**-cellulose diacetate (**PPIX@CA**) membrane by electrospinning. Reprinted with permission from Ref. [141]. 2021, Elsevier.

Recently, Ghiladi, Wang and co-workers [141], developed a porous photoactive material based on PPIX-embedded cellulose diacetate (**PPIX@CA**) microfibers (Figure 10). The photodynamic antimicrobial membrane was prepared by an electrospinning method with two different PPIX loadings: 5 and 10 wt.% PPIX with respect to CA (85 and 170 nmol PPIX/mg membrane, respectively). The antibacterial activity of the new material was evaluated towards Gram-(+) and Gram-(−) bacterial models. Despite the good efficacy of **PPIX@CA** porous microfibers against *S. aureus* after 30 min of white light irradiation (Xe lamp, λ ≥ 420 nm at an irradiance of 65 ± 5 mW cm^−2^), accomplishing 99.8% (~2.7 log) of bacterial reduction, the bacterial reduction observed for *E. coli* was lower (86.6%, ~0.87 log of reduction) using the same experimental protocol. The combined use of KI with the **PPIX@CA** porous microfibers showed to be beneficial since it further enhances the antimicrobial efficacy of the **PPIX@CA** microfiber membrane, achieving up to 99.9999% (6 log of reduction) photoinactivation of both *S*. *aureus* and *E*. *coli* strains in the presence of 25 and 100 mM KI, respectively.

#### 3.1.2. Hydroporphyrin Derivatives

Chlorins (e.g., chlorophylls) and bacteriochlorins (e.g., bacteriochlorophylls) are tetrapyrrolic macrocycles derived from porphyrins by the reduction of one or two diagonally opposite pyrrole ring double bonds, respectively [142].

Naturally occurring chlorophylls and bacteriochlorophylls are essential constituents of plants and bacteria, often called the *“pigments of life”* [134,135] owing to their important role in photosynthetic processes. Nowadays, their synthetic counterparts also present multiple applications essentially due to their absorbance of light in a broad range of the spectrum, particularly in the red (chlorins) and near infra-red region, NIR (bacteriochlorins) [65,143,144,145,146]. Intense red and NIR photons absorption is a desirable feature for biomedical applications (Imaging, PDT, aPDT), since the phototherapeutic window (650–900 nm) for electromagnetic radiation is the most penetrating and least harmful to human tissues [147,148]. Despite the interesting biological applications of natural semi-synthetic or synthetic hydroporphyrins, they are not naturally water-soluble and may aggregate in aqueous media [149,150]. Thus, several studies have been carried out to improve water solubility or dispersibility of hydroporphyrins and cellulose or cellulose-like supports have been employed as suitable carrier systems.

**Figure 11:**
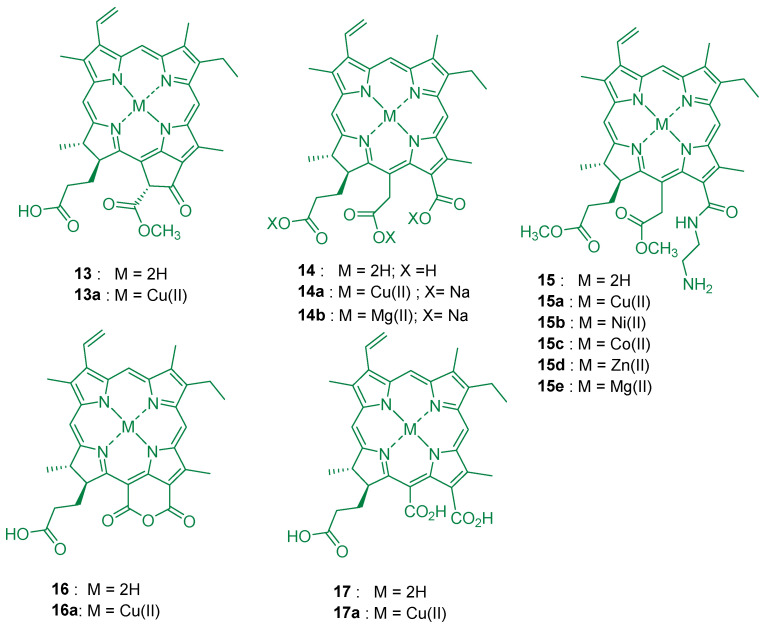
Structure of pheophorbide-*a* (**13**) chlorin *e_6_* (**14**), 6-*N*-(2-aminoethylamido)chlorin *e_6_* dimethyl ester (**15**) purpurin 18 (**16**) and chlorin *p*_6_, (**17**) and some metal complexes.

Despite not being used in microorganism inactivation, it is worthwhile to mention the seminal works of Roman and co-workers, where a hydroporphyrin, pheophorbide-*a* **13**, was recognized to have the potential to be combined with cellulose [151]. Solid samples of **13** (Figure 11) adsorbed on microcrystalline cellulose were prepared by adding weighed masses of cellulose to ethanol stock solutions of **13** (pheophorbide-*a* concentrations from 7.1 nmol to 8.9 μmol). After solvent evaporation by vacuum drying, the samples were studied and the absorption and luminescence properties of **13** adsorbed on cellulose support were investigated as a function of the dye concentration. 

As an extension of the previous work, the same authors [152] investigated the behavior of cellulose particles loaded with **13** in aqueous media.

The previously prepared samples of pheophorbide-*a*, **13**, adsorbed on microcrystalline cellulose (characterized in the solid state in previous work) were washed with water, leading to stable suspensions of micrometric particles (d < 2 μm) carrying photoactive and monomeric dye molecules. Suspensions were fluorescent and generated ^1^O_2_ efficiently. A similar effect has been observed on washing samples containing hematoporphyrin IX (HP) adsorbed on the same support. With this study, the authors conclude that, using cellulose as a heterogeneous carrier, it is possible to introduce hydrophobic PS into the aqueous medium while avoiding aggregation, thus preserving their photophysical properties. Results denote that cellulose can be employed as a suitable carrier system for **13**. It was also demonstrated that the same principle can be applied to carry HP into the water in its monomeric form. This fact opens the possibility of applying the same strategy to different hydrophobic PS. 

**Figure 12:**
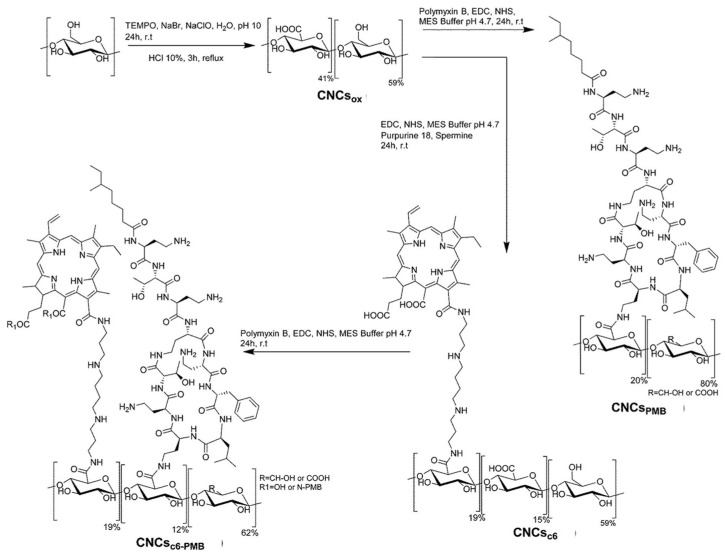
Synthetic route to cellulose nanocrystals **CNCs*c_6_*-_PMB_** coated with chlorin-*e6* and polymyxin B and to cellulose nanocrystals **CNCs_PMB_** just coated with polymyxin B. Reprinted with permission from Ref. [67]. 2017, The Royal Society of Chemistry.

In 2017, Sol and collaborators [67] developed a new strategy to devise photoactive material with effectiveness against infections caused by Gram-(−) multidrug resistant bacterial strains. A combination of two well-known concepts to eradicate microbes, aPDT and antibiotic prophylaxis, has been developed based on the fabrication of photobactericidal organic material, consisting of cellulose nanocrystals to which the purpurin 18 (**16**, see Figure 11) and the polypeptide polymyxin B (**PMB**) were covalently attached. The photoactive nanocrystals were prepared in three steps: (i) cellulose nanocrystals (CNC) were first obtained by oxidation of commercial cotton (with TEMPO as oxidant) to enable after hydrolysis **CNCs_ox_**, (Figure 12); (ii) purpurin 18 (**16**) was selected as PS and the reaction of cyclic anhydride with the free amine of spermine linker yielded a chlorin *e_6_* (**14**) bearing a linker able to form amide grafting group with nanocrystaline cellulose support CNCs*e_6_* (Figure 12); (iii) the antimicrobial **PMB** was covalently attached to the same cellulose support, yielding the photobactericidal organic material, **CNCsc_6_-_PMB_**, (Figure 12). In parallel, cellulose nanocrystals functionalized only with polymyxin B (**CNCs_PMB_**) were also synthesized, for comparison with the hybrid material bearing chlorin *e_6_* **CNCsc_6_-_PMB_** (Figure 12).

Following visible light irradiation (15 h at an irradiance of 0.8 mW cm^−2^), the photoactive material **CNCsc_6_-_PMB_** (0.191 mg mL^−1^) demonstrated high antibacterial efficiency (6 log units decrease) against Gram-(+) bacteria, *S. aureus* and *S. epidermidis*. A bacterial photoinactivation >6 log units was achieved against Gram-(−) bacterium *E. coli* when **CNCsc_6_-_PMB_** was exposed to the same light conditions (15 h, 0.8 mW cm^−2^) for both tested concentrations (0.191 mg mL^−1^ and 0.019 mg mL^−1^). Similar results of bacterial growth inhibition (>6 log of reduction in CFU/mL) were obtained for *P. aeruginosa* when the concentrations 0.191 mg mL^−1^ and 0.080 mg mL^−1^ of **CNCsc_6_-_PMB_** were used under light irradiation.

An amplifying and synergistic effect of chlorin *e_6_* has been highlighted against these Gram-(−) strains, based on membrane weakening and a potential docking effect from the polymyxin B moiety. Such results confirm the importance of using an antimicrobial peptide to broaden the spectrum of aPDT.

Later on, Gavara and coworkers [90] reported the possibility of utilizing copper(II) and magnesium(II) metal complexes of chlorin *e_6_* (**14a**, **14b** in Figure 11) incorporated in (hydroxypropyl)methyl cellulose (HPMC) for applications as photoactive coatings in polyethylene terephthalate (PET) packages. The antimicrobial properties of **14a** and **14b** in HPMC as coatings in PET packages were analyzed against *Listeria monocytogenes* and *E. coli.* The antimicrobial results indicated that **14b** [M = Mg(II)] was more active than **14a** [M = Cu(II)]) and that it was more effective against Gram-(+) bacterium *L. monocytogenes* (7 log reduction) than against *E. coli* where no significant growth reduction was observed.

### 3.2. Synthetic Meso-Substituted Porphyrins

#### 3.2.1. Neutral Porphyrins

**Figure 13:**
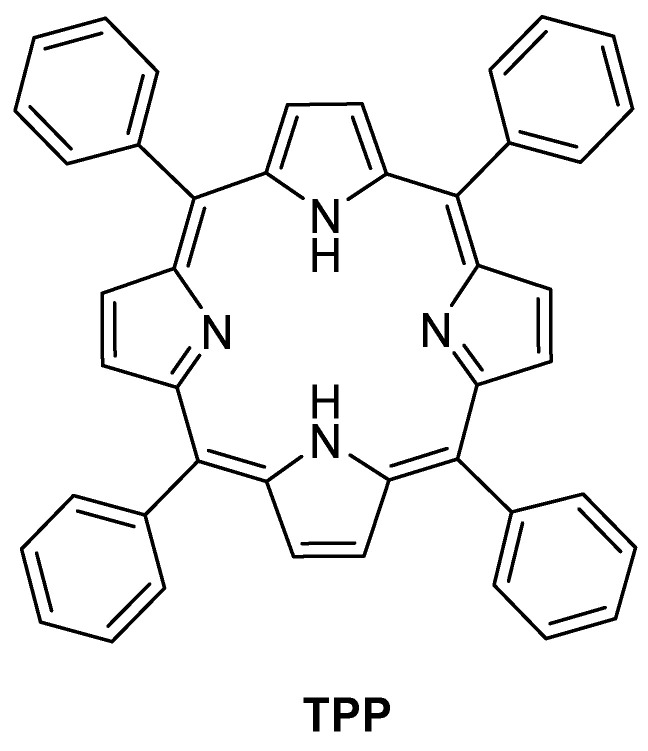
Structure of 5,10,15,20-tetraphenylporphyrin (**TPP**), the first *meso*-tetraarylporphyrin (tArPs) synthesized in laboratory.

Synthetic *meso*-tetraarylporphyrins (tArPs) are versatile compounds (Figure 13), readily accessible by facile and straightforward condensation reactions using pyrrole and aldehydes or dipyrromethanes/tripyrranes as starting materials [153,154]. Their chemical stability, photophysical features, and ability to fine-tune the electronic properties, makes tArPs a multipurpose platform for the preparation of materials with light-harvesting properties capable to generate ROS, namely ^1^O_2_ [76,155].

**Figure 14:**
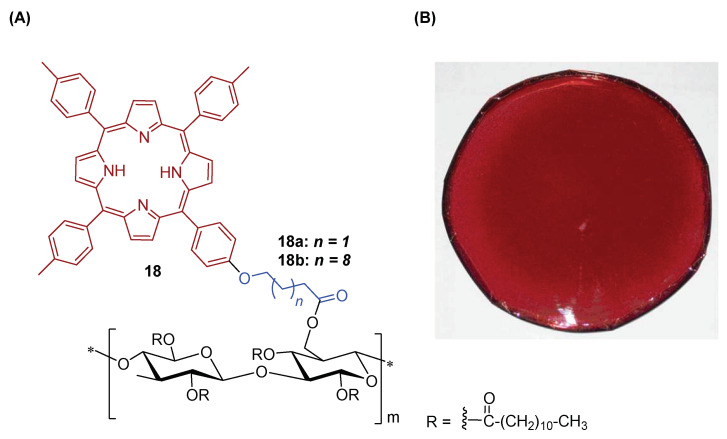
(**A**) Structure of tri-tolyl porphyrin derivative immobilized in ester laureate cellulosic polymer hybrid material (**18a** and **18b**) prepared by Krausz and co-workers (adapted from reference [156]). (**B**) Porphyrinic cellulose laurate ester plastic. Reprinted with permission from Ref. [156]. 2008, Elsevier.

One of the seminal works reporting a functionalized tArP derivative immobilized on cellulose support was reported by Krausz and co-workers [156], who developed photobactericidal plastic films by appending porphyrins through the acylation approach (Figure 14). Two series of porphyrinic cellulose laurate esters (**18a** and **18b**), with PS covalently linked to the cellulosic polymer, were prepared in “one-pot, two-steps” esterification reactions: (i) the tri-tolylporphyrin derivative was first covalently bounded to the cellulosic polymer and (ii) the porphyrin containing plastic films were then obtained by the second esterification with lauric acid (Figure 14). The antimicrobial properties of new photoactive cellulosic polymers were assessed against Gram-(+) and Gram-(−) bacterial strains (*S. aureus* and *E. coli,* respectively) under white light conditions (24 h irradiation at an irradiance of 1.7 mW cm^−2^) and the qualitative preliminary results are presented. The porphyrinic cellulose laurate esters **18a** and **18b** were found effective towards *S. aureus* and *E. coli* (no bacterial growth was observed under the films) when the grafting percentage of porphyrin was higher than 0.16% whereas the non-porphyrinic film control allowed full growth of bacteria. The biological data showed that the bacterial inactivation of the immobilized PS was mostly mediated by ^1^O_2_ which ultimately damages the cell wall since there is no penetration of the PS into the bacterial cell.

**Figure 15:**
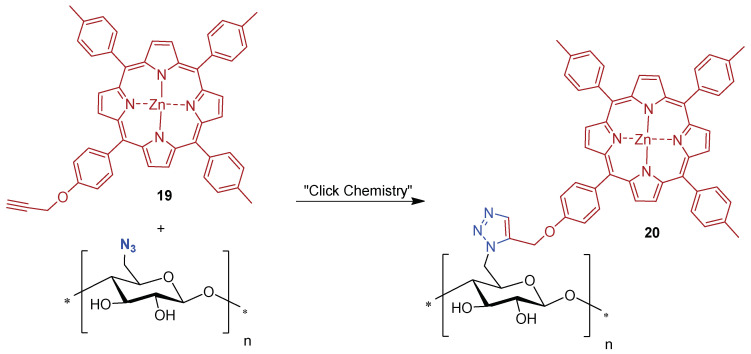
Synthetic methodology used to covalently graft complex **19** with a propynyloxy substituent in azido cellulose fabric. (adapted from reference [157]).

Later on, Krausz, Sol and co-workers [157] explored the *“Click-Chemistry”* synthetic approach to immobilize the Zn(II) complex **19** bearing a propynyloxy substituent on a cellulose cotton fabric functionalized with azide units (Figure 15). The new photoactive material **20** was characterized by ATR-FTIR and the antibacterial properties were evaluated. The authors showed for the fabric a similar antibacterial activity upon visible light irradiation against *E. coli* and *S. aureus* (reductions of ~5 log units).

**Figure 16:**
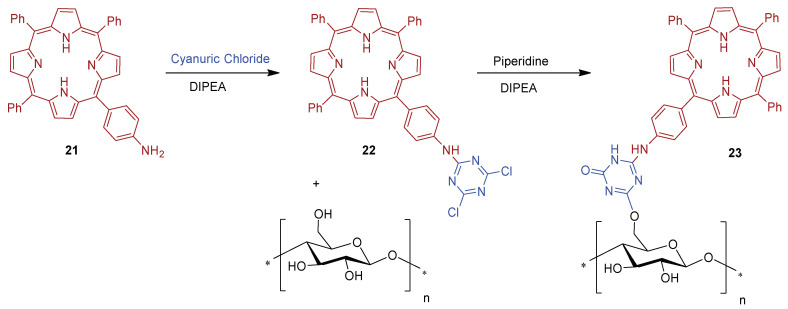
Synthetic methodology used to covalently graft 5-(4-aminophenyl)-10,15,20-triphenylporphyrin **21** on cellulose fabrics (cotton) via cyanuric chloride as grafting agent (Adapted from reference [158]).

Based on the versatility, reactivity, and availability for stepwise substitution of chlorine atoms by different nucleophiles, Sol and collaborators [158] used cyanuric chloride (2,4,6-trichloro-1,3,5-triazine), as a grafting agent between porphyrins and cellulose fabrics. The unsymmetric 5-(4-aminophenyl)-10,15,20-triphenylporphyrin derivative **21** was linked to triazine in the presence of a base, *N,N*-diisopropylethylamine (DIPEA) at 0 °C (Figure 16). Then, porphyrin-triazine **22** was grafted onto cellulose square fabrics, in this case using DIPEA plus piperidine, and was tested in vitro against *S. aureus*.

Experimental data showed that the cotton–PS material **23** produced a photobactericidal effect at quantity as low as ~0.8 μmol cm^−2^ of PS on the fabric sample. After 24 h exposure to white light and a total light dose of 9.5 J cm^−2^, a reduction of 93.7% (~1.2 log reduction) of bacterial abundance was achieved when compared with the initial bacterial concentration.

During the experiment, the bacterial growth increased by 4 logs for all the untreated (without PS) cotton samples either in the dark or under light irradiation. The same bacterial growth profile was observed for the cotton-PS material **23** in the dark (used as control). These controls demonstrated that either chemical modification of cotton alone or light exposure alone had no influence on bacterial reduction.

**Figure 17:**
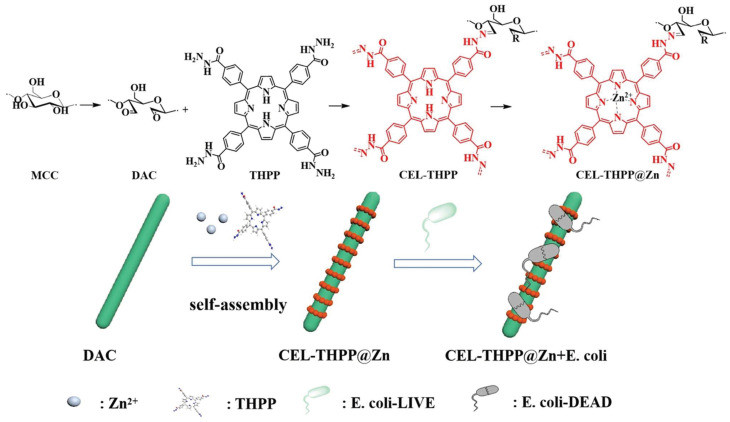
Synthetic methodology used to covalently graft porphyrin 5,10,15,20-tetrakis(4-hydrazinecarbonylphenyl)porphyrin (THPP) on oxidized cellulose containing aldehyde groups (DAC), Reprinted with permission from Ref. [159]. 2009, Springer Nature.

In 2022, Chen and collaborators [159] reported the development of the porous fiber **CEL-THPP@Zn** with promising antibacterial features (Figure 17). The authors used microcrystalline cellulose (MCC) to obtain, via oxidation with sodium periodate, the oxidized cellulose containing aldehyde groups (DAC), which was used to immobilize through imine bonds the porphyrin 5,10,15,20-tetrakis(4-hydrazinecarbonylphenyl)porphyrin (THPP). Then, the self-assembly of the resulting hybrid **CEL-THPP** in the presence Zn(II) ions afforded the desired composite **CEL-THPP@Zn** as a rod-shaped crystalline fiber loaded with porous porphyrin-zinc nanospheres. The antibacterial activity of the new prepared material was assessed against Gram-(+) *S. aureus* and Gram-(−) *E. coli* selected as bacterial models. A time-dependent correlation of bacterial inhibition as well as a correlation with antibacterial material concentration were found. The viability of *E. coli* incubated with 5 or 20 µg mL^−1^ concentrations of **CEL-THPP@Zn** decreases from 82% to 1%. In the case of *S. aureus,* a higher **CEL-THPP@Zn** concentration (200 µg mL^−1^) was needed to achieve total bacteria inactivation. The antibacterial behavior was justified considering the destruction of the bacterial membrane through the porphyrin zinc nanospheres. The ability of these porous fibers to adsorb both bacteria was also evaluated, opening the possibility of these composites to be used not only on bacterial inhibition, but also in filtration processes.

#### 3.2.2. Cationic Porphyrins

*Meso*-substituted porphyrins, bearing positively charged groups have shown remarkable photodynamic activity against both, Gram-(−) and Gram-(+) bacteria [66,81,160]. Positive charges are able to induce a strong electrostatic interaction with the external negatively charged structures of the bacterial cells, thus facilitating their interaction with the bacteria [161,162]. However, other PS properties, such as amphiphilicity, cell membrane interaction, and internalization, can also play a role to account for the therapeutic effect [161,163].

**Figure 18:**
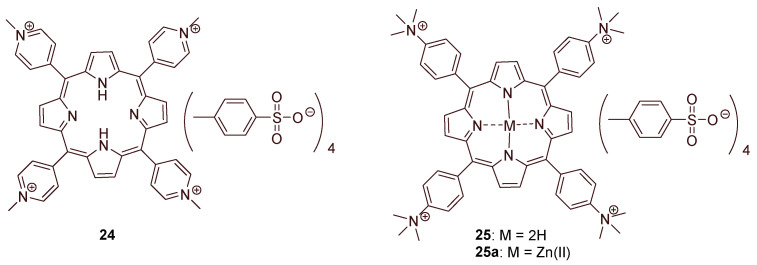
Tetracationic porphyrin derivatives used as PS in Bonnett and Galia studies [164].

In the pivotal work of Bonnett and Galia [164] (1994), regenerated cellulose (“*cellophane*” films) was considered as a low cost support for the impregnation of cationic porphyrins 5,10,15,20-tetrakis(1-methylpyridinium-4-yl)porphyrin tetra-tosylate (**24**) and 5,10,15,20-tetrakis(*N*,*N*,*N*-trimethylanilinium-4-yl)porphyrin tetra-tosylate (**25**) (Figure 18).

Commercially available *cellophane* sheets of 50 μm thickness were kept in contact with the porphyrin solutions of water or water/methanol at 50 °C. The uptake of positively charged porphyrin was estimated by UV-Vis spectroscopy after washing the cellophane films thoroughly. The authors confirmed that the films containing the cationic porphyrins **24** and **25**, after exposure to a 1500 W xenon lamp for 50 h, retained their mechanical properties (flexibility and strength) and were not photobleached. No bacterial growth was verified when the cellophane films impregnated with porphyrin **24** (18 μg cm^−2^) were maintained in contact with a bacterial inoculum of *S. aureus*, *Proteus vulgaris,* and *E. coli* for 24 h under visible white light (8W fluorescent lamp) irradiation.

Similar photobactericidal results were reported with the **25** cellophane films only towards *S. aureus*. The authors highlighted that the attempts to impregnate the cellophane films with anionic porphyrins were not successful.

**Figure 19:**
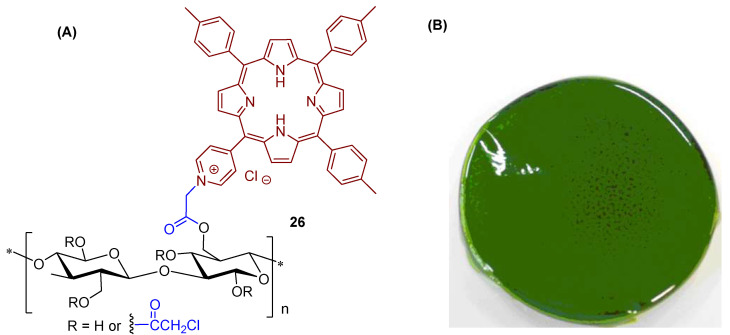
(**A**) Structure of material **26** used by Krausz and co-workers [165] as photobactericidal materials. (**B**) Porphyrinic plastic film prepared. Reprinted with permission from Ref. [165]. 2008 Elsevier.

Later on, Krausz and co-workers [165] prepared and characterized photobactericidal materials **26** through the immobilization of 5-(pyridin-4-yl)-10,15,20-tris(*p*-tolyl)porphyrin in chloroacetyl cellulose esters (Figure 19A). 

First, cellulose was dissolved in dimethylacetamide (DMAc)/LiCl and was reacted with chloroacetyl chloride leading to a functionalized plastic film with 2-chloroacetoxy units. The modified polymer was isolated by precipitation with water. Purification was carried out by successive dissolution–precipitation cycles and plastic films were then obtained by casting. The grafting of 5-(pyridin-4-yl)-10,15,20-tris(*p*-tolyl)porphyrin on chloroacetyl cellulose esters was achieved by a reaction of the plastic film previously synthesized, dissolved in anhydrous DMF, with the porphyrin derivative, at room temperature. The plastics films obtained after quaternization of the pyridine units were obtained by casting in a Petri dish after the adequate work-up (Figure 19B). 

The photoactive material **26** was tested against *E. coli* and *S. aureus* and the results showed that the high photobactericidal activity of the plastic films **26** prepared (no bacterial growth) was attributed to ^1^O_2_ generation and was dependent on the amount of porphyrin grafting (≥0.2 porphyrin content). In the absence of porphyrinic PS, the films allowed the full growth of bacteria.

**Figure 20:**
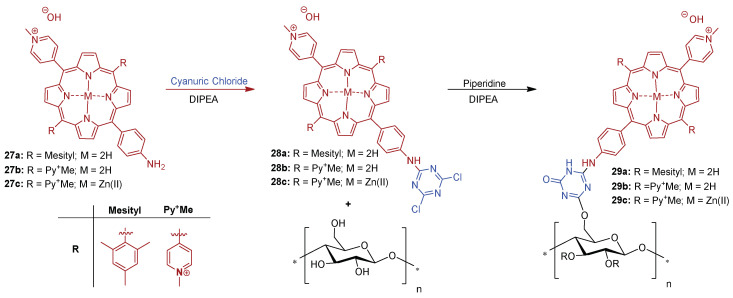
Synthetic methodology used to prepare the cellulose fabrics **29a**–**c** from cationic amino porphyrins using cyanuric chloride to introduce the grafting unit (Adapted from references [158,166]).

In 2010, the same group [158] reported the covalent grafting of a cationic amino porphyrin **27a** on cotton fabrics via a 1,3,5-triazine linker **28a** without any preliminary chemical modification of the cellulosic material (Figure 20). In fact, several research groups have explored 1,3,5-triazine linkers for the fabrication of covalently linked porphyrin-cellulose antimicrobial materials. The resulting porphyrin-triazine derivative grafted on cotton fabrics **29a** was tested in vitro against *S. aureus*. When the prepared cationic material **29a** was exposed to a white light for 24 h with a total light dose of 9.5 J cm^2^, a very high photoinactivation performance against *S. aureus* was achieved, with the limit detection of the method attained (>6 log of reduction).

The authors also assessed the efficacy of the photomaterial **29a** towards the gram-(−) bacterial strain *E. coli* [166]. After 24 h of exposure to white light (a total light dose of 9.5 J cm^−2^) of photomaterial **29a** and *E. coli*, no photoactivity was found. However, in the same photodynamic conditions, high growth inhibition rates of *S. aureus* (>4 log reduction) were reached. Despite the good results achieved for light activated PS, the material **29a** showed toxicity in the dark (80%) towards *S. aureus*. The authors attribute this fact to the presence of positively charged groups in the porphyrin macrocycle, known for their membrane disruption action on bacteria, even without light activation [166].

**Figure 21:**
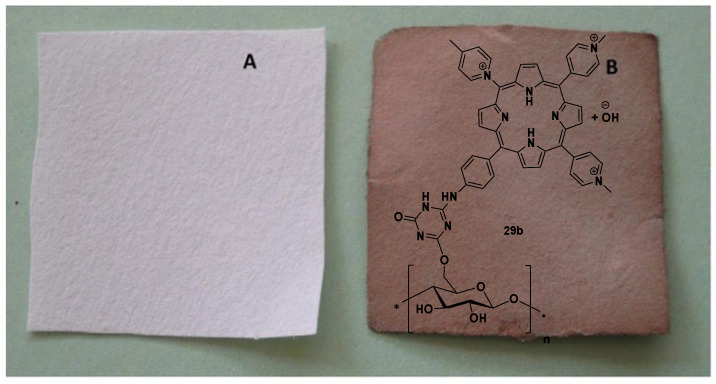
Photographs of (**A**) filter paper; (**B**) filter paper after reaction with **28b** giving **29b**. Reprinted with permission from Ref. [167]. 2012, Elsevier.

An extension of the previous studies was reported by the same research group [167]. The authors used the same approach (cyanuric chloride as a grafting agent) to immobilize **27b** in filter paper (Figure 21) and evaluated the antimicrobial efficacy of this bactericidal paper **29b** against *S. aureus* and *E. coli* bacterial strains. The authors showed that the untreated paper in the dark, or under white light irradiation, as well as the treated paper in the dark, allowed a bacterial growth of 4 and 2 log units for *S. aureus* and *E. coli*, respectively, when compared with the initial bacterial concentration. However, no survival of either type of bacteria was detected on the paper bearing the porphyrin **27b** after white light exposure for 24 h with a total light dose of 9.5 J cm^−2^ (inhibition of ~4 and 2 log units for *S. aureus* and *E. coli*, respectively).

**Figure 22:**
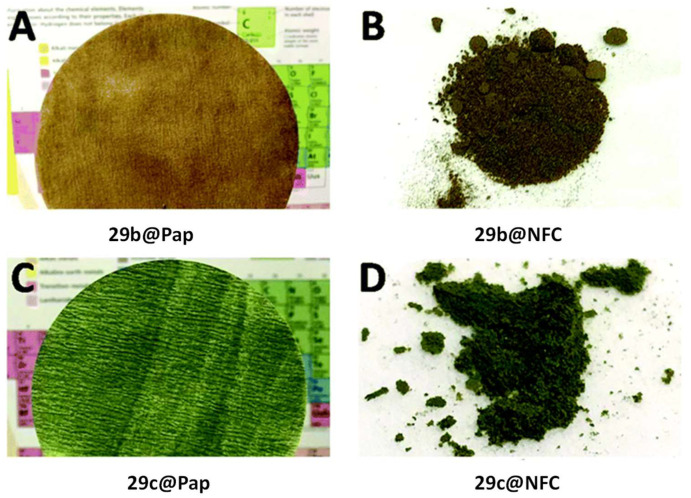
PS–cellulose conjugate materials (**A**) **29b@Pap**; (**B**) **29b@NFC**; (**C**) **29c@Pap**; (**D**) **29c@NFC**. Reprinted with permission from Ref. [125]. © The Royal Society of Chemistry 2019.

Studies conducted by Ghiladi group [125], reported the development of self-disinfecting materials through the covalent attachment of **27b** to nano-fibrillated cellulose (**NFC**) and paper (**Pap**) (Figure 22). The grafting strategy was centered on an environmentally sustainable method where the treatment of cellulose supports was mediated by an aqueous solution of cyanuric chloride, avoiding the use of organic solvents. Then, the **27b** and its Zn(II) complex **27c** were covalently attached by means of 1,3,5-triazine linkage to **NFC** or paper giving the materials **29b@NFC**, **29c@NFC**, **29b@Pap**, and **29c@Pap** (Figure 20).

In vitro aPDT assays employing the PS–cellulose conjugated materials (**29b@NFC**, **29c@NFC, 29b@Pap**, and **29c@Pap**) (Figure 22) were performed under fixed illumination conditions (1 h, white light (400–700 nm) at an irradiance of 65 ± 5 mW cm^−2^ towards four multiresistant bacteria strains (*S. aureus*; *Enterococcus faecium*, *Acinetobacter baumannii* and *Klebsiella pneumoniae*) and two enveloped viruses (Dengue-1 and vesicular stomatitis virus (VSV)). Table 1 summarizes the efficacy of prepared materials against the bacterial strains and viruses tested.

It was observed that against Gram-(+) bacteria, methicillin-resistant *S. aureus* (MRSA), and *E. faecium*, all photoactive materials achieved the detection limit of the inactivation method (99.9999%, 6 log unit reduction in CFU mL^−1^). In terms of killing Gram-(−) bacterium *A. baumannii*, most of the materials achieved the method detection limit (99.9999%, 6 log of abundance reduction) with exception of **29b@NFC** which was able to inhibit 4.5 log units (99.994% of reduction). For **29b@NFC**, no antibacterial activity was found against *K. pneumoniae* whereas photoinactivation with **29c@NFC** reached ~82% (0.73 log of reduction in CFU mL^−1^). On the other hand, when paper supports have been employed against *K. pneumoniae* photoinactivations of 99.9994% (~5.3 log of reduction) in the presence of **29b@Pap** and 99.47% (~2.3 log of reduction) for **29c@Pap** have been achieved.

The antiviral photodynamic inactivation studies employing **29b@NFC** and **29c@NFC** were conducted towards the two model enveloped viruses, (VSV and dengue-1), using similar photodynamic conditions to those of the antibacterial studies. Impressively, both materials **29b@NFC** and **29c@NFC** exhibited photoinactivation of the viral particles: dengue-1 by 99.99% (4 log reduction in PFU mL^−1^), and VSV by 99.9999% (6 log reduction in PFU mL^−1^). However, a dark viral inactivation of ~90% (~1 log of reduction in PFU mL^−1^) was also revealed in the dark controls.

**Figure 23:**
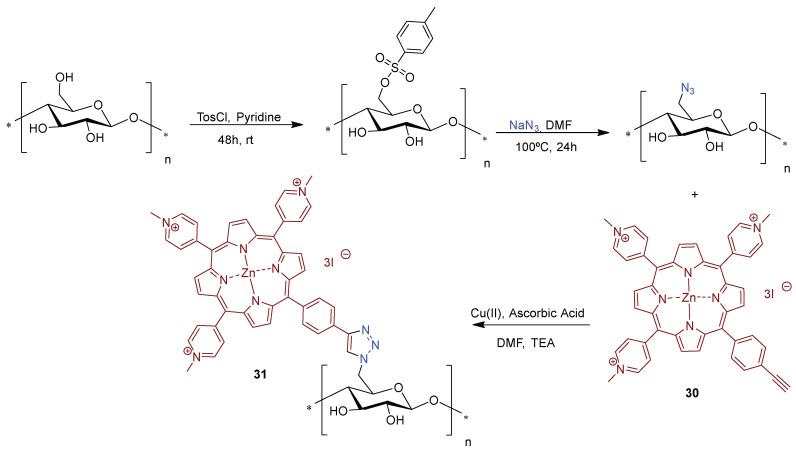
Ghiladi grafting method to prepare material **31** by covalently grafting the tricationic porphyrin **30** bearing an alkynyl function to cellulose nanocrystals or cellulose fibers (*Whatman #1* filter paper) bearing azide moieties (Adapted from Refs. [168,169,170]).

Ghiladi research group [168,169] also employed the “*Click Chemistry*” approach [Cu(I)-catalyzed Huisgen 1,3-dipolar cycloaddition] to immobilize covalently the tricationic porphyrin **30** bearing an alkynyl function to cellulose nanocrystals bearing azide moieties, giving material **31** (Figure 23). The authors prepared the cellulose nanocrystals by hydrolysis of *Whatman #1* filter paper (98% α-cellulose, 80% crystallinity) previously blended using aqueous HBr. After removing the excess of acid, water-soluble fragments and ultrafine particles were obtained with an average length of 100–400 nm. These nanocrystals were then functionalized with the azide moieties for further reaction with the alkynylporphyrin **30**. It was demonstrated that a suspension of material **31** showed an excellent photodynamic inactivation efficacy toward *Mycobacterium smegmatis* and *S. aureus*. However, only slight activity against *E. coli* was attained.

In the first aPDT assays, the bacterial cultures were irradiated with white light (400–700 nm at an irradiance of 60 mW cm^−2^) for 30 min (total light dose 108 J cm^−2^) in PBS with 20 µM of **31** after a dark incubation period and the results showed a 6 log reduction in viable cells against *S. aureus* (5 min incubation), 3.5 log for *M. smegmatis* (45 min incubation), and 2 log for *E. coli* (60 min incubation) [168].

As an extension of the previous work, in order to gain insight into the photodynamic action mechanism, the same group used the same photoactive material against different bacterial strains [169]. In that view, photoactive material **31** (20 µM) was used in the inactivation of multidrug resistant *A. baumannii*, *S. aureus* and *Pseudomonas aeruginosa* (≈10^8^ CFU mL^−1^) with visible light at an irradiance of 65 mW cm^−2^ [169]. A decrease of 6 log in viable cells was observed for *S. aureus* (5 min incubation), 5–6 log units for sensitive *A. baumannii* (30 min incubation) and multidrug resistant *A. baumannii* (15 min incubation) and 2.5 log units for *P. aeruginosa* (60 min incubation) after receiving a total light dose of 118 J cm^−2^ (30 min of irradiation). Confocal laser scanning microscopy analysis of samples of *A. baumannii* or *S. aureus* incubated with the photoactive material **31** suggested a lack of PS internalization by the bacteria. Considering both studies, the authors commented that material **31** was able to mediate the photodynamic inactivation of all the tested bacterial strains studied. Confocal microscopy demonstrated that the mode of action for **31** did not proceed through a PS-binding or uptake mechanism. The cytotoxic species (e.g., ^1^O_2_ and other ROS) generated by the photodynamic process damaged the bacteria wall, leading to cell inactivation. These results corroborate other studies that highlight the inactivation efficiency of porphyrins towards bacteria without PS internalization requirement [161].

Considering the promising results, and having in mind scalability for the production of anti-infective or self-sterilizing materials, Ghiladi and co-authors [170] also employed cellulose paper activated with azide moieties to graft other porphyrin derivatives (neutral and negatively charged) and BODIPYs bearing an alkynyl unit via the “*Click*-*Chemistry*” approach. The bactericidal papers obtained with a dye load of ca. 8 mg g^−1^ (12.4 nmol mg^−1^) were fully characterized and their antibacterial efficacy was evaluated against “ESKAPE” pathogens, e.g., *S. aureus*, vancomycin-resistant *E. faecium*, *A. baumannii*, *P. aeruginosa*, and *K. pneumoniae*. However, from all the bactericidal papers prepared, the best results were obtained with the material **31** (positive charged porphyrin derivative) that was able to photoinactivate >4 log (99.99%) of all bacterial strains studied upon illumination for 30 min with white light (400−700 nm) at an irradiance of 65 mW cm^−2^. The same material **31** was also able to photoinactivate dengue-1 virus by >4 log, influenza A virus by ~2.5 log (~99.5%), and human adenovirus-5 by ~2 log (~99%).

**Figure 24:**
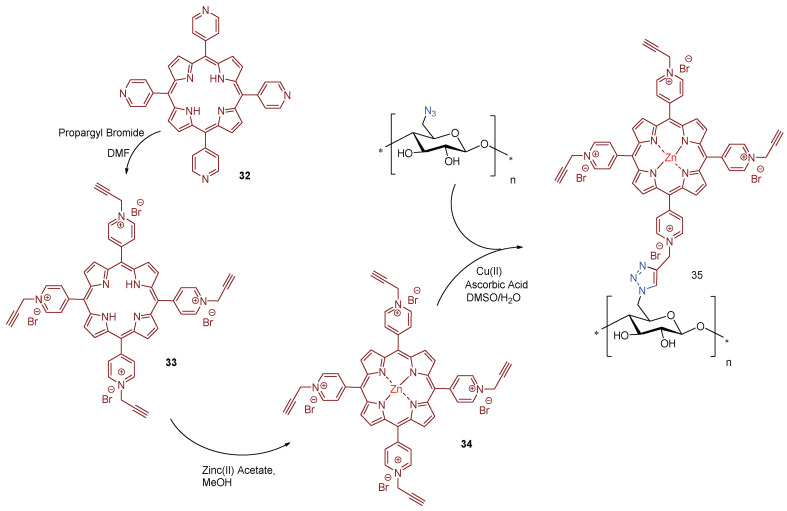
Synthetic strategy adopted by Zerrouk and co-workers to obtain the propargylated cationic porphyrin **34** and to graft it into the azide kraft pulp (adapted form [171]).

Zerrouk research groups [171] also used the concept of “*click chemistry*”, as a strategy for grafting propargylated cationic porphyrins in azide modified cellulose materials. Zerrouk considered Kraft Pulp (KrP) as cellulose raw-material for the preparation of antimicrobial photoactive material. The material was first functionalized, via hydroxyl group tosylation, following by the azide group insertion in the presence of sodium azide, yielding 6-azido-6-deoxycellulose (Figure 24). Then, the treatment 5,10,15,20-tetra(pyridin-4-yl)porphyrin **32** with an excess of propargyl bromide at room temperature afforded the propargylated porphyrin derivative **33** in quantitative yield that after coordination with Zn(II) afforded the corresponding metalloporphyrin **34**.

The covalent linkage between the azide kraft pulp and the propargylated cationic metalloporphyrin **34** was carried out using Cu(I)-catalyzed alkyne-azide cycloaddition (CuAAC) approach. The photodynamic antimicrobial activity of new cellulose material **35** was evaluated against the *S. aureus, E. coli*, and *P. aeruginosa* bacterial strains under white light irradiation (400–800 nm) at an irradiance of 0.16 mW cm^−2^ and a total light dose of 13.8 J cm^−2^. The functionalized kraft pulp precursors, untreated, tosylated, and azidated were used as control materials and no dark or light antibacterial activity was achieved for all controls. Furthermore, material **35** did not show activity against Gram-(−) bacteria *E. coli* and *P. aeruginosa* under dark and light conditions. However, bacteriostatic activity against Gram-(+) bacterium *S. aureus* was observed (99% of abundance reduction, 2 log reduction in CFU mL^−1^) after dark exposure to the material **35**, which changed to a bactericidal effect of (>99.9% of abundance reduction; 3 log reduction) under light conditions.

**Figure 25:**
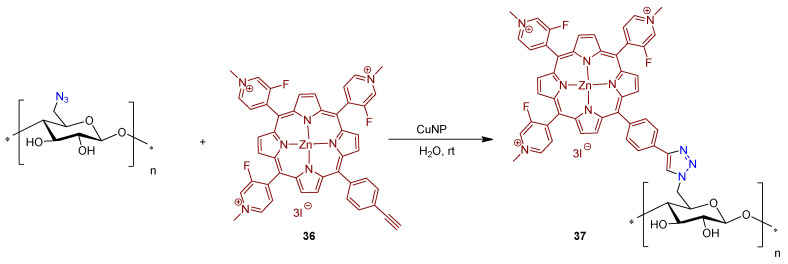
Synthesis of cellulose fluoro triazole-porphyrin conjugate **37** by “*click chemistry*” approach (Adapted from Ref. [172]).

A similar approach was used by Guesmi to obtain material **37** from the mono-propargylated cationic fluoroporphyrin **36** and cellulose functionalized with azide units (Figure 25) [172]. The catalytic coupling reaction was performed in water and in the presence of copper nanoparticles (CuNP). 

Although photodynamic inactivation was not accessed in this study, the antimicrobial activity of the newly porphyrin-cellulose material **37** was evaluated in the dark after incubation with *E. coli* and *S. aureus*, along with the non-functionalized textiles as a comparison, at different times. The results showed that the bactericidal efficacy was time dependent: after 20 min of bacterial strain incubation with material **37**, a 3 log (99.9%) reduction on bacterial growth was noticed, whereas a 6 log (99.9999%) reduction was obtained after 80 min of photodynamic treatment.

In addition, in 2016, Rahimi, Rassa and Fayyaz [104,173] reported the antimicrobial properties of 5,10,15,20-tetrakis(*N,N,N*-trimethylanilinium-4-yl)porphyrin, **25** and of its Zn(II) complex **25a** after being just embedded in cellulosic fabrics. The process involved the soaking of a 100% cellulosic fabric, pre-treated with an aqueous solution of Na_2_CO_3_, with solutions of porphyrins **25** and **25a** in PBS for 30 min at 50 °C. After this period, the unbound porphyrin was thoroughly washed, and the molar embedding ratio was calculated by UV-Vis. The authors reported that the embedding yield was dependent on the initial concentration of the PS and its presence in the cellulosic fabrics was confirmed by spectroscopic techniques. The antimicrobial activity of the new materials was tested under visible light irradiation (100 W tungsten lamp with an average irradiance of ~0.36 mW.cm^−2^) towards *S. aureus*, *P. aeruginosa*, and *E. coli*. The results showed that the irradiation period and the PS concentration were important factors to be taken into account. *S. aureus* was full inactivated (>3 log reduction) with both prepared cellulose fabrics at PS concentration of 100 µM after 30 min of irradiation while for *P. aeruginosa* (20% of reduction), at the same PS concentration, the full inactivation (>3 log reduction, >99.9%) was only attained in the presence of Zn(II) complex **25a** after 90 min of irradiation. The worse efficiency of these materials was observed towards *E. coli* (58.5% for **25** and 30% for **25a**, <0.4 log reduction) at the same concentration and after 90 min of irradiation. 

#### 3.2.3. Anionic Porphyrins

The above-mentioned section clearly demonstrates that aPDT research efforts have been focused on the development of cationic complexes, as their biocide efficacy arises from the possibility of interaction of these compounds with the bacterial cell membrane. The development of anionic porphyrins, regardless of their high efficiency to produce ROS, does not receive great attention because of their low interaction on the membrane. Anionic porphyrins carry typically sulfonated [174,175,176,177], carboxylic acid/carboxylate [178,179] or phosphonate/phosphinate groups [180,181,182]. These negatively charged functional groups induce an inherent solubility of the porphyrins in aqueous media, which would greatly benefit their application in biological or environmental contexts.

Although there are several reports dealing with the immobilization of cationic and neutral porphyrins onto cellulosic materials, to date, there have been only two studies that describe the covalent linking of an anionic porphyrin derivative onto a cellulose scaffold and its use in light-promoted inactivation of bacteria.

**Figure 26:**
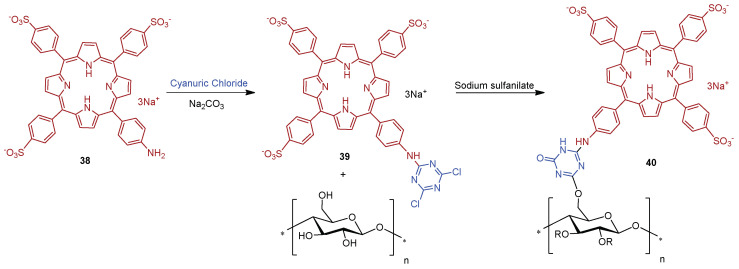
Synthetic methodology used to covalently graft the anionic amino porphyrin **38** on cellulose fabrics via cyanuric chloride approach (Adapted from reference [158]).

In this context, Sol and co-workers [158] developed a study where the anionic porphyrin 5-(4-aminophenyl)-10,15,20-tris(4-sulfonatophenyl)porphyrin trisodium **38** was compared with **27a** and **27b** (Figure 20) counterparts for aPDT application. The anionic derivative **38**, after reaction with cyanuric chloride, afforded porphyrin **39**, which was then grafted on a cotton surface through 1,3,5-triazine linkage, yielding material **40** (Figure 26).

The photodynamic efficacy of anionic porphyrin-grafted in cotton **40** was tested in vitro, against *S. aureus*. When functional cellulose-based material **40** (~10 μmol of PS) was incubated for 24 h and exposed to white light with a total light dose of 9.5 J/cm^2^, the bacterial reductions were 37% (~0.2 log reduction), 94.7% (~1.3 log reduction), and >99.99% (>4 log reduction) for anionic, neutral, and cationic materials, respectively. The experimental data suggest that results are dependent on the PS charge. It was highlighted that the cationic material **29a** showed the highest antimicrobial effect against *S. aureus*, probably due to the presence of a positive charge, responsible for disrupting cell walls.

Later on, Carofiglio and co-workers [183] explored the features and properties of the photoactive material obtained by conjugating the anionic and water-soluble amino porphyrin **38** with nanocrystalline cellulose (NCC). 

**Figure 27:**
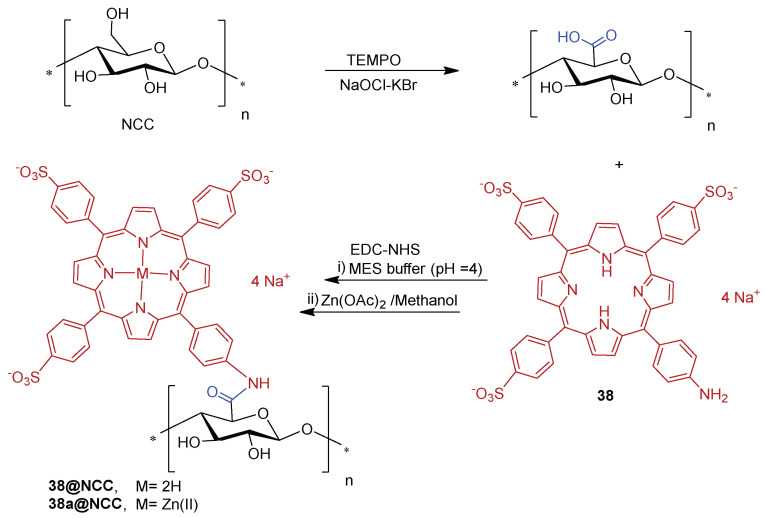
Synthetic methodology used to covalently graft via an amide bond porphyrin 5-(4-aminophenyl)-10,15,20-tris(4-sulfonatophenyl)porphyrin trisodium **38** on oxidized commercial microcrystalline cellulose. (Adapted from reference [183]).

The NCC support selected was produced by sulfuric acid treatment of commercial microcrystalline cellulose (66% of crystallinity). The oxidation with TEMPO in the presence of NaOCl–NaBr selectively converted some primary hydroxyl groups on the NCC surface to carboxylic functionalities. The conjugation of **38** to the obtained NCC-COOH via amide linkage (Figure 27) was performed using carbodiimide crosslinker chemistry in an acidic aqueous solution of 2-(*N*-morpholino)ethanesulfonic acid (MES, 50 mM, pH = 4) in the presence of *N*-(3-dimethylaminopropyl)-*N*-ethylcarbodiimide hydrochloride (EDC.HCl) and *N*-hydroxysuccinimide (NHS). The **38@NCC** material was isolated after three days under stirring, followed by centrifugation, and washing work-up. Additionally, the authors evaluated the potential of this photoactive material as PS by evaluating the ^1^O_2_ generation. However, no photoinactivaton studies against bacteria have been presented until now. 

## 4. Other Cellulose Based Biopolymer and Other Cellulose-like Fibres as Supports

**Figure 28:**
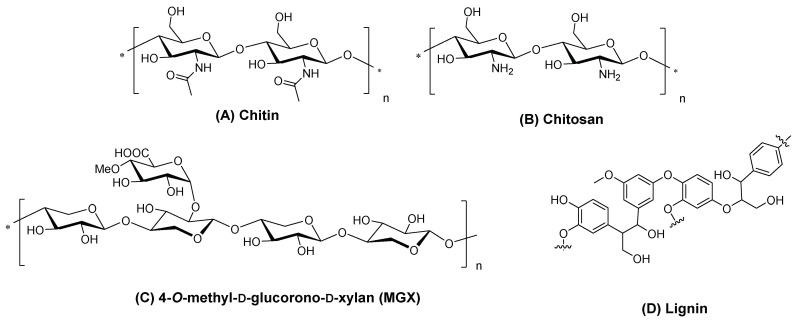
General structures for biopolymers also considered as supports in this review.; (**A**) Chitin, (**B**) Chitosan; (**C**) Xylan; (**D**) Lignin.

In addition to cellulose, other non-toxic and biodegradable supports, such as chitosan, chitin, xylan or lignin biomaterials (Figure 28), and organic synthetic polymers as supports for PS to be used in aPDT, deserve to be discussed since they have an impact in this research field [184,185].

In the grafting of PS to synthetic polymers, different approaches could be considered: (i) covalent or non-covalent linking of the macrocycle with polymers, or (ii) the *co*-polymerization of PS with polymerizable moieties with monomers or electrospinning techniques. 

**Figure 29:**
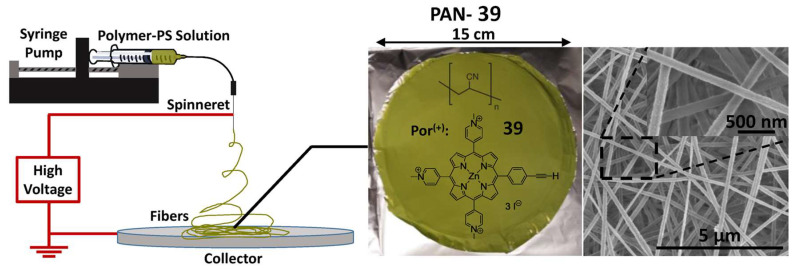
Electrospinning schematic (**left**), PAN-**39** (middle), and scanning electron microscopy (SEM) images (**right**). Reprinted with permission from Ref. [186]. © 2022 Frontiers Media S.A. All rights reserved.

The quest for affordable and industrially robust fabrication technologies of antimicrobial surfaces and biomaterials led Ghiladi and co-workers [186] to employ electrospinning in the preparation of a non-woven textile comprised of polyacrylonitrile nanofibers (PAN) embedded with a porphyrin-based cationic photosensitizer **39**, termed PAN-**39** (Figure 29). Antibacterial efficacy of PAN-**39** material (PS loading 34.8 nmol mg^−1^ of material) was evaluated against multiresistant bacterial models: *S. aureus*; vancomycin-resistant *E. faecium*; *A. baumannii*; *K. pneumonia*, as well as *E. coli*. The results obtained showed a broad photodynamic inactivation of all multiresistant bacterial strains studied upon white light exposure (400–700 nm, 30 min of exposure at an irradiance of 65 ± 5 mW cm^−2^) by a minimum of 99.9996% (>5.8 log of abundance reduction) regardless of taxonomic classification. PAN-**39** also inactivated human adenovirus-5 (~99.8% reduction in PFU/mL, ~3 log reduction) and VSV particles (>7 log reduction in PFU mL^−1^). When compared to previous studies with cellulose-based materials employing this same PS (porphyrin **30**) [168,169,170], the higher photodynamic inactivation performance achieved here with PAN-**39** is likely due to the combined effects of higher PS loading and a greater surface area imparted by the use of nanofibers. 

**Figure 30:**
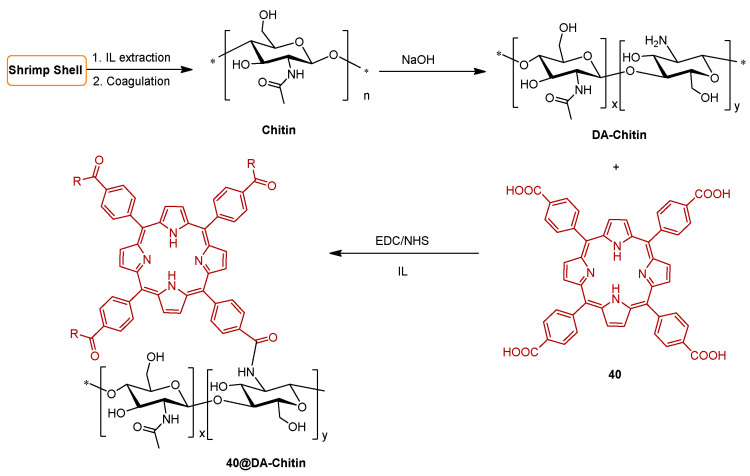
Strategy used by Rodgers to immobilize 5,10,15,20-tetrakis(4-carboxyphenyl)porphyrin (**40**) on chitin support, using ionic liquids (Ils) as solvents for both, chitin extraction and deacetylation (adapted form Ref. [187]).

The work of Rodgers and collaborators [187] consisted in the immobilization of 5,10,15,20-tetrakis(4-carboxyphenyl)porphyrin **40** on a chitin support (the second most abundant natural polysaccharide, after cellulose, a linear polymer composed of repeating β(1→4)-*N*-acetylglucosamine units) using ionic liquids (ILs) as solvent. In this study, the Ils were used for chitin extraction and partial deacetylation, as well as a medium for grafting **40** on chitin support. Chitin was first extracted from shrimp shells using the 1-ethyl-3-methylimidazolium acetate ([C_2_mim][OAc]), coagulated in water, and then deacetylated. The deacetylated-chitin (**DA-chitin**) was dissolved in [C_2_mim][OAc] and the covalent attachment between the amino groups of **DA-chitin** and the carboxyl groups of **40** was performed in the presence of the activators EDC and *N*-hydroxy-succinimide (NHS) (Figure 30). The resulting polymeric composites were cast as a film and coagulated with water to remove the IL and the excess of reagents, resulting in homogeneous **40@DA-chitin** films. Further modifications of the optical properties were achieved by the incorporation of metal ions (Cu^2+^, Zn^2+^, Gd^3+^, and Fe^3+^) into the **40@DA-chitin** films. In addition, the **40@DA-chitin** films, with and without chelating metal ions, exhibited efficient ^1^O_2_ production under light irradiation.

Since these films showed interesting optical and photophysical properties (good ^1^O_2_ generation and tunable optical properties), the authors anticipated that such films have a great potential to be used in aPDT.

**Figure 31:**
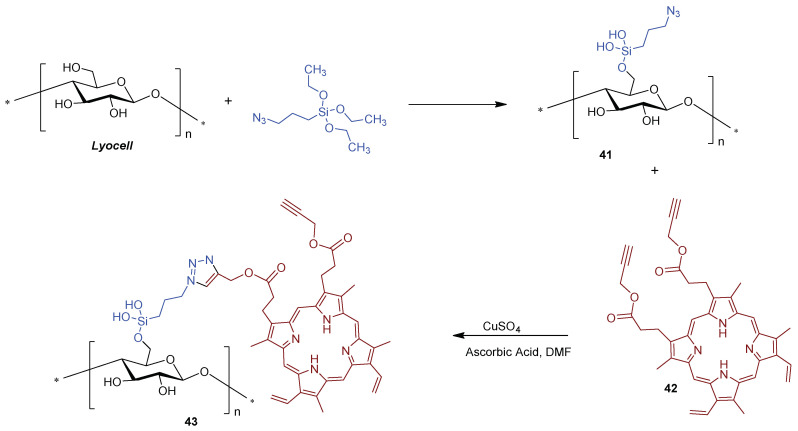
Synthetic route to immobilize alkynylated PPIX **42** on alkoxysilane azido-modified *Lyocell* fibers **41** (adapted from Ref. [121]).

Hetteger and co-workers [121] selected a cellulose-based fiber, namely *Lyocell*, to support porphyrins according to the azide-alkyne “*click*” concept (Figure 31). First, azido-modified *Lyocell* fibers **41** and alkynylated PPIX **42** building blocks were prepared through alkoxysilane chemistry and Steglich esterification (DCC/4-dimethylaminopyridine), respectively. *Lyocell* fibers were pre-activated by swelling in organic solvents to increase the accessibility of hydroxyl groups in the subsequent silanization process. Then, both building blocks were combined under the “*Click Chemistry*” approach affording photoactive *Lyocell* fibers **43** (Figure 31) which after a full characterization, by FTIR, NMR, UV/Vis spectroscopy, and elemental analysis, was biologically screened. The photobactericidal activity of the photoactive *Lyocell* fibers **43** against *S. aureus* and *B. subtilis* was confirmed by the diminishing bacterial growth rate of both Gram-(+) strains. These achievements corroborated the potential envisaged for these fibers as antimicrobial textiles to be applicable at home, hospitals, and health care facilities.

**Figure 32:**
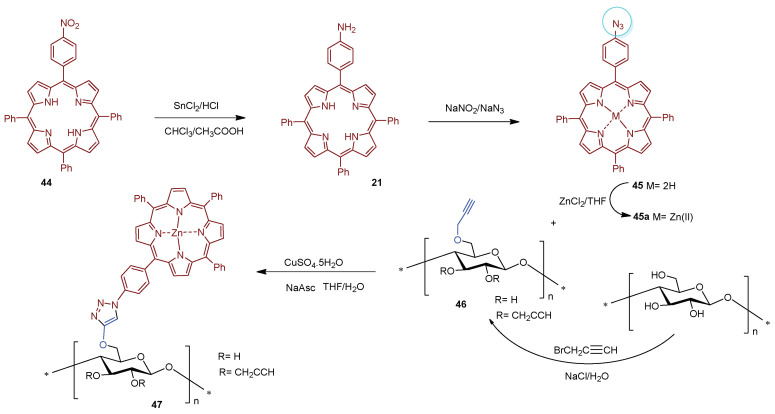
Synthetic route for the preparation of metalloporphyrin **45a** and conditions used for its grafting on modified cellulose **46** (adapted from Ref. [130]).

Following the same *“Click Chemistry”* approach, Zerrouki and co-workers [130] grafted the Zn(II) complex of 5-(4-azidophenyl)-10,15,20-triphenylporphyrin **45a** onto Kraft pulp fibers. The Kraft pulp material was modified with propargyl bromide giving propargylated pulp fibers **46** (Figure 32). The functionalized support **46** was characterized by FTIR to evaluate the presence of C-C triple bond and by XPS to determine the degree of substitution. The 5-(4-azidophenyl)-10,15,20-triphenylporphyrinatozinc(II) **45a** was prepared in four sequential steps: (i) condensation of pyrrole with nitrobenzaldehyde and benzaldehyde, giving 5-(4-nitrophenyl)-10,15,20-triphenylporphyrin **44**; (ii) reduction of nitro group with Sn(II) chloride in acidic media, affording the 5-(4-aminophenyl)-10,15,20-triphenylporphyrin **21**; (iii) diazotization of the aminoporphyrin **21** in the presence of NaN_3_ affording the azidoporphyrin 5-(4-azidophenyl)-10,15,20-triphenylporphyrin **45**, and, finally, (iv) metalation with Zn(II) acetate in THF. The Zn(II) metalloporphyrin complex **45a** was grafted by the azide moiety on the propargylated Kraft pulp fibers **46** using a copper(I)-catalyzed coupling reaction, giving the photoactive Kraft fiber material **47**.

The antimicrobial activity of material **47** was tested under visible light irradiation against *S. aureus* and *P. aeruginosa* used as Gram-(+) and Gram-(−) bacterial models, respectively. The two bacterial strains deposited on the photosensitizing Kraft pulp material **47** were efficiently killed (no surviving bacteria were detected, against to 4.5 log in the controls) after 24 h exposure to white light (total light dose of 9.5 J cm^−2^).

Abdelraouf and Senge [188] recently developed a photoactive material based on the loading of 5,10,15,20-tetrakis(3-hydroxyphenyl)porphyrin (3-THPP) onto an ethylcellulose (EC)/chitosan (Chs) nanocomposite. Chitosan is an amino polysaccharide, produced from the deacetylation of chitin. The obtained conjugate involving 3-THPP and the nanocomposite EC/Chs was characterized by different techniques as FT-IR, SEM, EDX, XRD, DLS, and UV–NIR absorption. The authors compared the photodynamic activity of 3-THPP before and after being embedded on the nanocomposite (3-THPP vs. 3-THPP@EC/Chs). When the non-supported 3-THPP was irradiated with laser light in the blue region of the electromagnetic spectrum (458 nm, 488 nm, and 476 nm; power of 70 mW) for only 15 min, reductions of 83.6 ± 1.2%, 83.4 ± 0.5%, and 87.0 ± 0.9% (<1 log reduction) in the microbial survival rates of fungus *Candida albicans* and bacterial strains of *P. aeruginosa* and *S. aureus* were achieved, respectively. A lower performance was observed for 3-THPP@EC/Chs material when irradiated in the same blue light conditions, with microbial survival rates reductions of 56.1 ± 0.7%, 59.2 ± 0.9%, and 55.5 ± 0.8%, respectively.

On the other hand, when 635 nm (red light) was chosen to irradiate 3-THPP@EC/Chs (15 min at 5 mW), the microbial survival rates of *C. albicans*, *P. aeruginosa*, and *S. aureus* was lower than in the presence of free 3-THPP.

**Figure 33:**
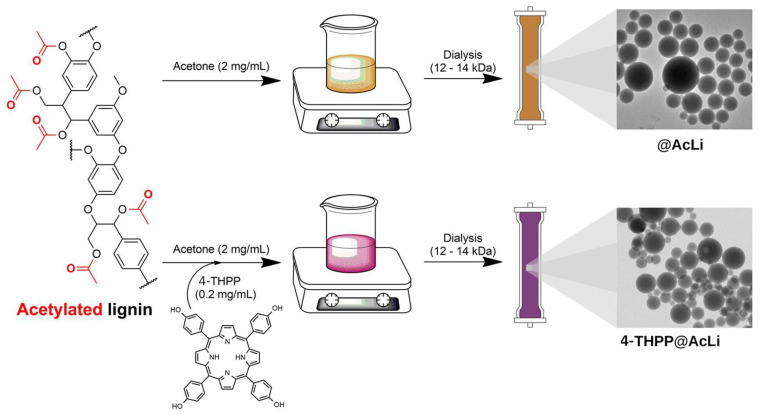
Preparation of acetylated lignin nanoparticles (AcLi) and 5,10,15,20-tetrakis(4-hydroxyphenyl)porphyrin (**4-THPP**)-loaded on acetylated lignin nanoparticles (**4-THPP@AcLi**). Reprinted with permission from Ref. [189]. 2022, Frontiers Media S.A.

In 2020, Leroy-Lhez and co-workers [189] used acetylated lignin nanoparticles (AcLi) to encapsulate 5,10,15,20-tetrakis(4-hydroxyphenyl)porphyrin (4-THPP) (Figure 33). It was shown that this system (**4-THPP@AcLi**) was effective to produce ROS and presented a photodynamic effect against the three Gram-(+) strains tested (*S. aureus*, *S. epidermidis*, and *Enterococcus faecalis*), being capable to reduce bacterial abundance in 99.9% (3 log reduction) when exposed to white light and a total light dose of 4.16 J cm^−2^, with 4-THPP concentrations below 5.0 μM.

Despite the significant photodynamic action against Gram-(+) bacteria, the newly prepared material **4-THPP@AcLi** has not shown any antimicrobial activity against, Gram-(−) bacteria (*E. coli* and *P. aeruginosa*). The authors also proved by TEM, once again, that nanoparticles did not penetrate inside the bacterial cell, suggesting that the inactivation effect was elicited on the outside of bacterial wall.

The results of aPDT accomplished by Leroy-Lhez and co-authors motivated the same authors to extend the strategy on the preparation of photoactive material with acetylated lignin containing other porphyrin derivatives. In 2022 [190], they reported the encapsulation of 5,10,15,20-tetrakis(4-hydroxyphenyl)porphyrin and four other porphyrin derivatives (5,10,15,20-tetrakis(4-acetyloxyphenyl)porphyrin; 5,10,15,20-tetrakis(4-hydroxyphenyl)porphyrinatozinc(II), 5,10,15,20-tetrakis(4-(3-(*N,N,N*-trimethylammoniumpropoxy)phenyl)porphyrin tetra-bromide and 5,10,15,20-tetrakis(1-(3-hydroxypropyl)pyridinium-4-yl)porphyrin tetra-bromide) inside acetylated lignin nanoparticles (AcLi).

Although all these porphyrin derivatives were successfully encapsulated, the resulting acetylated lignin nanoparticles disclosed a limited photodynamic bactericide effect under blue-LED light, 450–460 nm, 15 J cm^−2^ on *S. aureus* and *E. coli*. 

Having in mind the possibility of using the aPDT approach to prevent food spoilage and deterioration or to be used in a clinical context, Castro and collaborators [61,179] considered and developed photoactive materials using porphyrin derivatives incorporated into chitosan films in view of having a high degree of reusability, without any great loss of photodynamic efficacy. 

**Figure 34:**
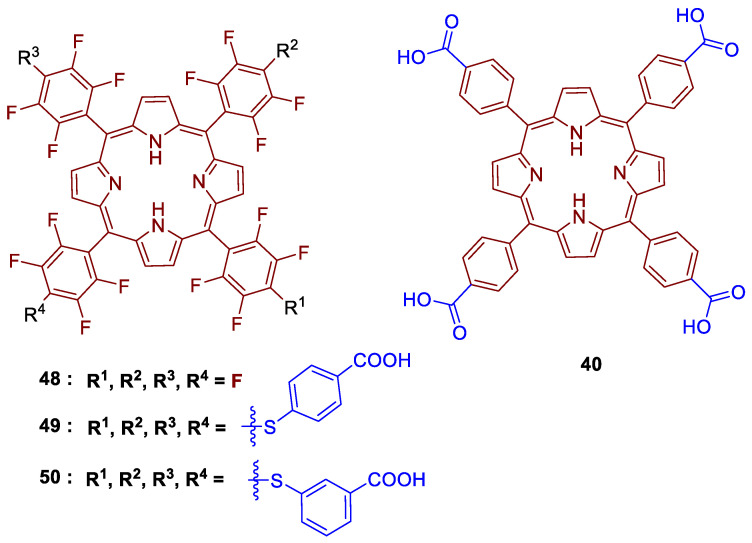
Porphyrinic derivatives **40**, **48**, **49**, **50** immobilized on chitosan. Adapted from Ref. [61].

Aiming to prepare new materials for preventing the development of bacterial biofilm in the food industry, the authors prepared a set of photoactive antimicrobial films obtained by the grafting of 5,10,15,20-tetraarylporphyrins in chitosan with different functionalities, e.g., phenyl units bearing carboxyl groups or pentafluorophenyl units without or with 3- or 4-mercaptobenzoic acid groups, as shown in Figure 34 [61]. The efficiency of these antimicrobial films was assessed on the prevention of *Listeria innocua* attachment and consequent biofilm formation under different irradiation protocols. *L. innocua* biofilm growth was nearly completely inhibited in two of the porphyrin-chitosan films, one containing the 5,10,15,20-tetrakis(pentafluorophenyl)porphyrin **48** and the other the thio-carboxylate porphyrin **50** after being exposed to white light (at an irradiance of 10 mW cm^−2^) for 24 h followed by incubation in the dark for 48 h.

**Figure 35:**
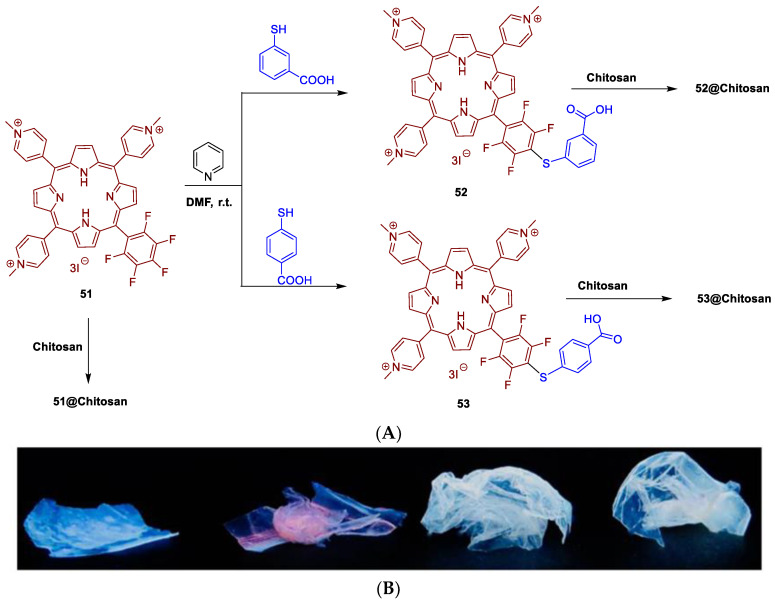
(**A**) Synthesis of porphyrinic derivatives **51**, **52**, **53** and their immobilization on chitosan giving **51@chitosan**, **52@chitosan** and **53@chitosan**. (**B**) From left to right: Images of **chitosan**, **51@chitosan**, **52@chitosan** and **53@chitosan** under ultraviolet light. Reprinted with permission from Ref. [179]. 2019, MDPI, Basel.

Later on, the same research group functionalized 5,10,15-tris(1-methylpyridinium-4-yl)-20-(pentafluorophenyl)porphyrin tri-iodide **51** with 3- or 4-mercaptobenzoic acid, yielding two new tricationic porphyrins, **52** and **53**, with suitable carboxylic pending groups to be incorporated on chitosan (Figure 35).

The cationic porphyrins **51**, **52** and **53** were immobilized on chitosan by adding each porphyrin dissolved in THF to a solution of chitosan in an aqueous acetic acid solution. 

The new materials **51@chitosan**, **52@chitosan**, and **53@chitosan** were evaluated under white light towards the recombinant bioluminescent *E. coli* (3.0 µM under 3.0 mW cm^−2^ of white light irradiation) The most effective material against *E. Coli* was **51@chitosan**, which showed a bacterial inactivation of 4.1 log reduction in CFU/mL (>99.99% of reduction) after 90 min of irradiation. In comparison, when the bacteria were exposed to **52@chitosan** and **53@chitosan**, the bacterial reduction was almost negligible (decrease of 0.63 (76%) and 0.31 log (~50%) reduction in CFU/mL, respectively). The authors concluded that these porphyrins, in general, have lost their capacity to produce ^1^O_2_ when entrapped on a chitosan material.

## 5. Cellulose Based Photoactive Materials for Application in Cancer Therapy

As mentioned above, in addition to bacterial infections, the photodynamic approach can be applied with success to treat other diseases or to improve health conditions. In fact, photodynamic therapy (PDT) is a clinically approved treatment for oncologic, non-oncologic diseases, and tumor diagnoses [191]. Similarly to aPDT, it is based on the interaction of a photosensitizing dye (PS), light, or dioxygen, and involves the production of cytotoxic ROS, which trigger a cascade of reactions, leading to the destruction of abnormal cells [192]. The main advantages of PDT compared with traditional oncological therapies are due to its spatio-temporal nature, relatively non-invasive nature, lower systemic toxicity, relatively selective destruction of undesired cells, good cosmetic outcome, and its ability to stimulate the immune system, as well as its good tolerability profile [77,193].

However, PDT has also some limitations, e.g., the poor solubility of PS agents in biological media, which led the scientific community to search for better delivery systems. PS for PDT can become more efficacious when adsorbed, entrapped, or linked to cellulose-like supports which are characterized by biocompatibility and biodegradability [194]. In addition, these cellulose-based systems provide an increased surface area, a very interesting feature for a platform targeted drug delivery [195].

**Figure 36:**
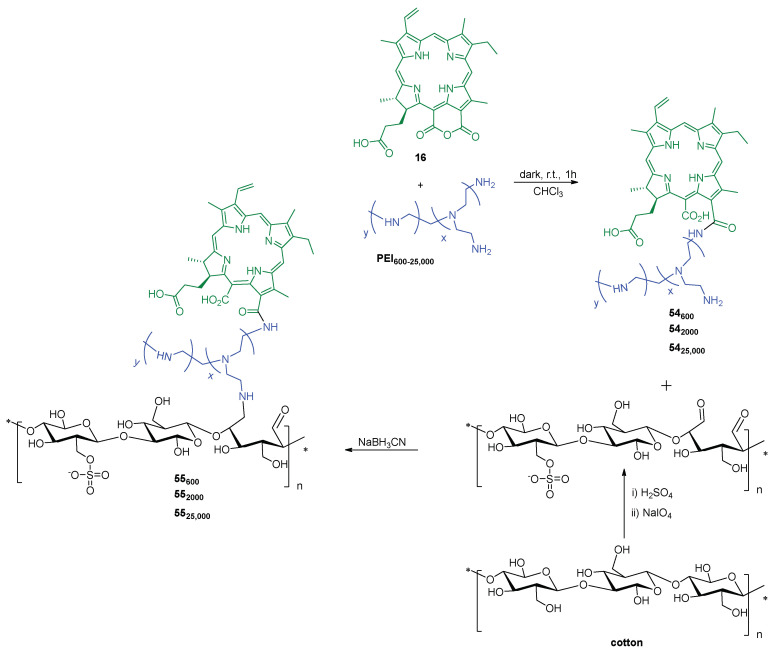
Synthetic methodology adopted by Sol and co-workers to fabricate new cellulose nanocrystals materials (**55_600-25,000_**), bearing purpurin 18 (**16**), conjugated with polyethyleneimine scaffolds (adapted from Ref. [68]).

In 2012, Sol and collaborators [68], with a view to improving tumor selectivity and cancer cell targeting of PSs, reported the synthesis, characterization, and biological applications of cellulose nanocrystal materials prepared from three different lengths polyethyleneimine (PEI) scaffolds (**54_600_, 54_2000_**, and **54_25,000_**) with purpurin 18 (**16**) (Figure 36).

In parallel, nanocrystalline cellulose material was obtained by the hydrolysis of cellulose (cotton) with sulfuric acid/sodium periodate treatment, to covalently attach the amino groups of **54** to the resulting carbonyl groups under reductive conditions (Figure 36).

The cytotoxicity potential of the new photoactive materials **55_600-25,000_** was evaluated in the dark on a human keratinocyte cell line (HaCat) and compared with Photofrin II^®^ used as control. The results showed that the photoactive material **55_600_** (PEI 600 Da) displayed the lowest dark cytotoxicity, close to that of Photofrin II^®^. Photocytotoxicity studies revealed the best IC_50_ (7.4 nM) for photoactive material **55_600_** towards HaCat cell line when compared with the 23 nM for Photofrin II^®^, showing that material **55_600_** is a very active PS in a nanomolar concentration range. Further studies demonstrated that the photodynamic action of **55_600_** induced apoptosis in a large fraction of treated HaCat. This cell death pathway is considered an advantage because apoptosis allows tumor cell destruction and subsequent elimination by phagocytosis.

Moreover, nanoparticles (NP) have been used as drug carriers, demonstrating an outstanding ability to improve PS drug delivery [196]. The encapsulation in NP of PSs with low solubility in water can enhance the overall delivery to targeted sites, improving biological properties such as tissue distribution and absorption rates.

**Figure 37:**
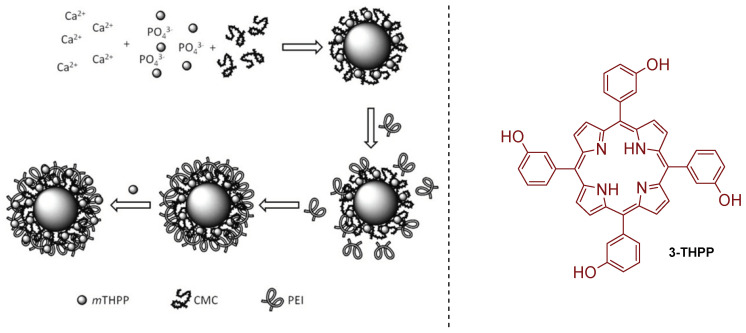
Schematic representation of NP containing calcium phosphate core with shells of carboxymethyl cellulose (CMC, anionic), poly(ethyleneimine) (PEI, cationic) and 5,10,15,20-tetrakis(3-hydroxyphenyl)porphyrin (**3-THPP**). Reprinted with permission from Ref. [197]. 2009, Springer Nature.

Under this context, Epple and co-workers [197] prepared a positively charged hybrid NP consisting of a calcium phosphate core and an organic shell. The calcium phosphate hybrid NP was prepared by precipitation from water and then functionalized with a mixture of 5,10,15,20-tetrakis(3-hydroxyphenyl)porphyrin (**3**-**THPP**) and the biocompatible anionic polymer carboxymethyl cellulose (CMC). The negative charge of the inner shell was then reversed by adding an outer shell of the positively charged polymer, polyethyleneimine (PEI) encapsulating the photoactive dye **3-THPP** (Figure 37). The hybrid NP, carrying the photoactive dye, was dispersed in water and the photodynamic activity against HT29 cells (human colon adenocarcinoma cells), HIG-82 cells (rabbit synoviocytes), and J774A.1 cells (murine macrophages) was accessed. A high photodynamic activity together with a very low dark toxicity was observed for both cell lines HIG-82 and J774A.1 at a concentration of 2.0 μM of **3-THPP**. This killing efficiency was equivalent to the non-immobilized **3-THPP**. The authors ascribed the high efficacy observed for the new materials to the positive charge of the NP surface (facilitating the transport through the negatively charged cell membrane) and to the high loading of **3-THPP** into the NP.

**Figure 38:**
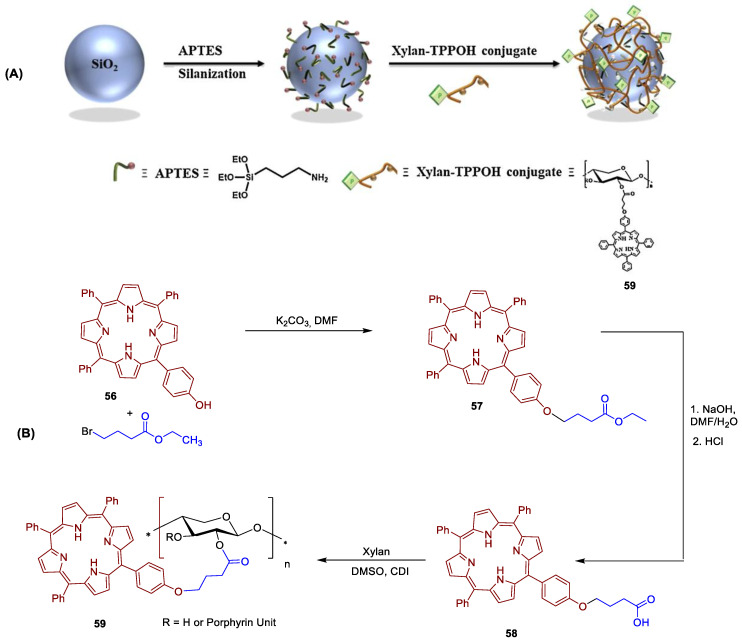
(**A**) Schematic procedure for the coating of silica NP with **56**. Reprinted with permission from Ref. [198]. 2008, Elsevier. (**B**) Synthetic route used to immobilize 5-(4-hydroxyphenyl)-10,15,20-triphenylporphyrin **56** on Xylan (adapted from Ref. [198]).

The enhancement of PS solubility and the improvement of photodynamic activity observed using a core-shell hybrid NP formed by a silica core and xylan NP was corroborated by Chaleix and co-workers in 2019 [198]. To afford drug delivery control and to increase the drug bioavailability on blood circulation, 5-(4-hydroxyphenyl)-10,15,20-triphenylporphyrin **56** was functionalized with ethyl 4-bromobutyrate through nucleophilic substitution giving **57**. After ester hydrolysis, in a basic medium, the resulting compound **58** was then covalently attached to xylan by esterification using 1′1’-carbonyldiimidazole (CDI) as a coupling reagent to afford the material **59** (Figure 38). Finally, **59** was used to coat the silica NP (SNP) affording **59@SNP** (Figure 38). The obtained nanomaterials were characterized and their therapeutic potential for PDT was evaluated against colorectal cancer cell lines (HCT116 and HT-29 cell lines). In vitro studies showed that **59@SNP** was 40-fold and 10-fold more effective against HCT116 cells and HT-29 cells, respectively, compared to free porphyrin **56**. The authors stated that, at a cellular level, the conjugation of **56** to SNPs (**59@SNP**) significantly enhanced its solubility and, consequently, the uptake by cancer cells.

## 6. Conclusions

This review intends to cover the literature data regarding the immobilization of porphyrin-like molecules on cellulose-based supports. We discussed the typical methods employed for: cellulose modification, functionalization of porphyrins by inserting groups allowing immobilization, as well as the synthetic routes to prepare the photosensitizer-cellulose functional materials. The new photoactive hybrid materials reported herein demonstrate unique properties, including improved mechanical strength and rheological features. These characteristics are excellent for medical applications, such as wound healing photoactive patches to treat diabetic feet, pressure ulcers, or burn wounds, but also for the antimicrobial photoinactivation of multiresistant bacteria in vast clinical contexts (surfaces, gloves, catheters, fabrics). The technological approach discussed herein for photosensitizer-cellulose functional materials offers the possibility to recover and reuse the photosensitizing system in a sustainable manner.

Concerning the application of photoactive cellulose as material in the field of aPDT, its effectiveness has clearly been proven in several contexts. Nonetheless, there still is room for new improvements, maximizing the full potential for translational research and forefront development.

Infections perpetrated by multiresistant bacteria are a current major concern. Improving the PS properties in terms of amphiphilicity, cell membrane interaction, and internalization are indeed important features to exert an effective therapeutical effect. These issues might be addressed by the development of new PS structures, but also through the development of new formulations and site-directed drug delivery cellulose. 

We hope that this review might contribute towards providing new insights regarding PS-graphed cellulose and motivate medical and scientific communities to devote further efforts to this topic.

## Figures and Tables

**Table 1:** Photodynamic antimicrobial and antiviral efficacies, of prepared materials (**29b@NFC**, **29c@NFC** and **29b@Pap**, **29c@Pap**).

	29b@NFC (Log CFU mL^−1^)	29c@NFC (Log CFU mL^−1^)	29b@Pap (Log CFU mL^−1^)	29c@Pap (Log CFU mL^−1^)
MRSA	6	6	6	6
*E. faecium*	6	6	6	6
*A. baumanii*	4.5	6	6	6
*K. pneumoniae*	*	0.73	5.3	2.3
Dengue-1 virus	4	4	nd **	nd **
VSV	6	6	nd **	nd **

aPDT Protocol conditions: irradiance of 65 ± 5 mW cm^−2^; white light (400–700 nm); 60 min of irradiation; PS concentration of 5.0 μM. * no statistically significant inactivation; ** not determined.
